# Noncanonical SQSTM1/p62-Nrf2 pathway activation mediates proteasome inhibitor resistance in multiple myeloma cells via redox, metabolic and translational reprogramming

**DOI:** 10.18632/oncotarget.11960

**Published:** 2016-09-10

**Authors:** Irene Riz, Teresa S. Hawley, Jeffrey W. Marsal, Robert G. Hawley

**Affiliations:** ^1^ Department of Anatomy and Regenerative Biology, George Washington University, Washington, DC, USA; ^2^ Flow Cytometry Core Facility, George Washington University, Washington, DC, USA; ^3^ Flow Cytometry Core, National Cancer Institute, National Institutes of Health, Bethesda, MD, USA

**Keywords:** multiple myeloma, carfilzomib, SQSTM1/p62, NFE2L2/Nrf2, translation initiation

## Abstract

Multiple Myeloma (MM) is a B-cell malignancy characterized by the accumulation of clonal plasma cells in the bone marrow, with drug resistance being a major cause of therapeutic failure. We established a carfilzomib-resistant derivative of the LP-1 MM cell line (LP-1/Cfz) and found that the transcription factor NF-E2 p45-related factor 2 (Nrf2; gene symbol *NFE2L2*) contributes to carfilzomib resistance. The mechanism of Nrf2 activation involved enhanced translation of Nrf2 as well as its positive regulator, the autophagy receptor sequestosome 1 (SQSTM1)/p62. The eukaryotic translation initiation factor gene *EIF4E3* was among the Nrf2 target genes upregulated in LP-1/Cfz cells, suggesting existence of a positive feedback loop. In line with this, we found that siRNA knockdown of eIF4E3 decreased Nrf2 protein levels. On the other hand, elevated SQSTM1/p62 levels were due at least in part to activation of the PERK-eIF2α pathway. LP-1/Cfz cells had decreased levels of reactive oxygen species as well as elevated levels of fatty acid oxidation and prosurvival autophagy. Genetic and pharmacologic inhibition of the Nrf2-*EIF4E3* axis or the PERK-eIF2α pathway, disruption of redox homeostasis or inhibition of fatty acid oxidation or autophagy conferred sensitivity to carfilzomib. Our findings were supported by clinical data where increased *EIF4E3* expression was predictive of Nrf2 target gene upregulation in a subgroup of patients with chemoresistant minimal residual disease and relapsed/refractory MM. Thus, our data offer a preclinical rationale for including inhibitors of the SQSTM1/p62-Nrf2 pathway to the treatment regimens for certain advanced stage MM patients.

## INTRODUCTION

Multiple myeloma (MM) is a B cell malignancy characterized by the clonal expansion of neoplastic plasma cells in the bone marrow. The introduction of novel agents such as proteasome inhibitors and immunomodulatory drugs has prolonged the lives of patients with MM over the past decade [1]. The first-in-class proteasome inhibitor bortezomib provided proof-of-principle that proteasome inhibition is an important therapeutic target in MM, leading to its FDA approval as frontline therapy in June 2008 [2]. Despite overall response rates of 100% in the setting of newly diagnosed disease [3], most MM patients ultimately relapse because the MM cells develop resistance to the treatment [4, 5]. Carfilzomib, a second-generation proteasome inhibitor that exhibits enhanced selectivity for the proteasome [6], received full approval from the FDA for the treatment of patients with relapsed/refractory MM in January 2016 [7]. However, the overall response rate to carfilzomib in the pivotal phase 2 clinical trial was less than 25% [8]. Furthermore, in a subsequent phase 3 study — in which overall survival was the primary endpoint [9] — carfilzomib monotherapy failed to significantly improve the survival of relapsed/refractory MM patients compared to those who received best supportive care (10.2 months *vs* 10.0 months) (ClinicalTrials.gov Identifier: NCT01302392). These results indicate that the majority of MM cells that became resistant to bortezomib were also resistant to carfilzomib. Clearly, to extend the life expectancy of patients with this disease, it is essential to characterize the mechanisms conferring resistance to proteasome inhibitors.

To begin to understand the underlying processes that might be relevant to clinical carfilzomib resistance in MM, we previously established carfilzomib-resistant derivatives of MM cell lines, KMS-11/Cfz and KMS-34/Cfz [10, 11]. In both cases, prosurvival autophagy was shown to contribute to carfilzomib resistance mediated, in part, *via* transcriptional upregulation of the *SQSTM1* gene encoding sequestosome 1/p62 (SQSTM1/p62) [11]. SQSTM1/p62 is a multifunctional scaffold protein that interacts with various signaling molecules and serves as a ubiquitin-binding cargo receptor connecting the proteasomal and autophagic protein degradation pathways [12]. Another important function of SQSTM1/p62 is activation of the transcription factor nuclear factor-erythroid 2 (NF-E2)-related factor 2 (Nrf2; gene symbol *NFE2L2*) in the Keap1-Nrf2 signaling pathway [13, 14]. The Keap1-Nrf2 pathway maintains cellular redox homeostasis by inducing antioxidant and detoxification genes and by modulating energy metabolism [15]. A growing body of evidence implicates activation of the Keap1-Nrf2 pathway as a contributor to therapy resistance [16] but, to our knowledge, its role in conferring resistance to carfilzomib in MM cells has not been described.

The Keap1-Nrf2 pathway is tightly regulated at multiple levels, the precise details of which are currently being elucidated [17]. Under basal conditions, Nrf2 monomers are sequestered in cytoplasmic complexes through two-site binding to Keap1 homodimers which target Nrf2 for continuous ubiquitination and proteasomal degradation [18]. In response to oxidative stress, the Keap1 molecules are modified such that ubiquitination of Nrf2 is inhibited and it remains bound to the complex [19, 20]. In a variation on this theme, overexpressed SQSTM1/p62 binds to and competitively prevents Keap1 from interacting with one of the Nrf2 sites, effecting a conformational change in the complex which leads to impaired ubiquitination of associated Nrf2 [13, 14]. In both scenarios, newly synthesized Nrf2 escapes cytoplasmic sequestration by Keap1 and is free to translocate to the nucleus and regulate gene expression. Of relevance in this regard, a number of reports have indicated that Nrf2 expression is subject to diverse mechanisms of translational control [21–25].

Nrf2 activity can also be modulated by other mechanisms [26–29]. For example, nuclear translocation and activation of Nrf2 is enhanced by phosphorylation by the PERK protein kinase [26, 27]. PERK is activated upon accumulation of misfolded/unfolded proteins in the endoplasmic reticulum which results in the induction of an “unfolded protein response” (UPR) [30]. The best characterized function of PERK on UPR induction is to provide a protective advantage to the cell by attenuating global protein translation *via* inhibitory phosphorylation of eukaryotic translation initiation factor-2α (eIF2α) [31, 32]. Phosphorylation of eIF2α also results in the preferential translation of certain mRNAs containing upstream open reading frames (uORFs), the prototypical example of which is activating transcription factor 4 (ATF4) [33]. During this phase of the UPR, ATF4 and Nrf2 coregulate transcription of some cytoprotective genes [34, 35]. However, if proteostasis is not restored, ATF4 induces a cell death program involving the CCAAT/enhancer-binding protein homologous protein (CHOP) transcription factor [36].

Here we report the establishment of a new carfilzomib-resistant derivative of the LP-1 MM cell line, LP-1/Cfz, in which carfilzomib resistance was characterized by induction of prosurvival autophagy as well as Nrf2 pathway activation associated with elevated SQSTM1/p62 levels. Unlike carfilzomib-resistant KMS-11/Cfz and KMS-34/Cfz cells, increased SQSTM1/p62 levels were not due to transcriptional upregulation of the *SQSTM1* gene [11]. Rather, the higher levels of SQSTM1/p62 were due to increased translation dependent in part on activation of the PERK-eIF2α axis. Comparative analysis with KMS-11/Cfz cells revealed Nrf2 target gene induction as well but only LP-1/Cfz cells were sensitized to carfilzomib by inhibition of the PERK-eIF2α signaling cascade. Additionally, LP-1/Cfz cells exhibited increased Nrf2 synthesis associated with elevated expression of Nrf2 targets involved in translation initiation, in particular, *EIF4E3* encoding an atypical eukaryotic translation initiation factor family member recently demonstrated to mediate context-specific translation in diffuse large B-cell lymphoma [37, 38]. Moreover, gene set enrichment analysis (GSEA) of gene expression profiling data from MM patient samples showed that increased *EIF4E3* expression was predictive of Nrf2 activation in some chemoresistant minimal residual disease and relapsed/refractory MM cases. These findings have elucidated further complexities of the proteostasis network in MM cells and provide preclinical rationale for therapeutic development of SQSTM1/p62-Nrf2 inhibitors as a means to overcome proteasome inhibitor resistance in a subgroup of advanced stage MM patients.

## RESULTS

### GSEA identifies Nrf2 pathway activation in carfilzomib-resistant MM cell lines

The carfilzomib-resistant LP-1/Cfz cell line was established by continuous culture of the LP-1 MM cell line [39] in stepwise increasing concentrations of the drug (4 nM to 12 nM) over an 18 week period according to a previously published protocol used to derive the carfilzomib-resistant MM cell lines, KMS-11/Cfz and KMS-34/Cfz [11] (Figure S1). As observed for KMS-11/Cfz and KMS-34/Cfz cells, LP-1/Cfz cells retained resistance to carfilzomib even when tested after removal of selective pressure for approximately 8 weeks. Gene expression profiling was performed on LP-1/Cfz and parental LP-1 cells, and GSEA was used to identify differentially overrepresented pathways and processes associated with carfilzomib resistance shared between LP-1/Cfz cells and the KMS-11/Cfz and KMS-34/Cfz cell lines [11].

**Figure 1:**
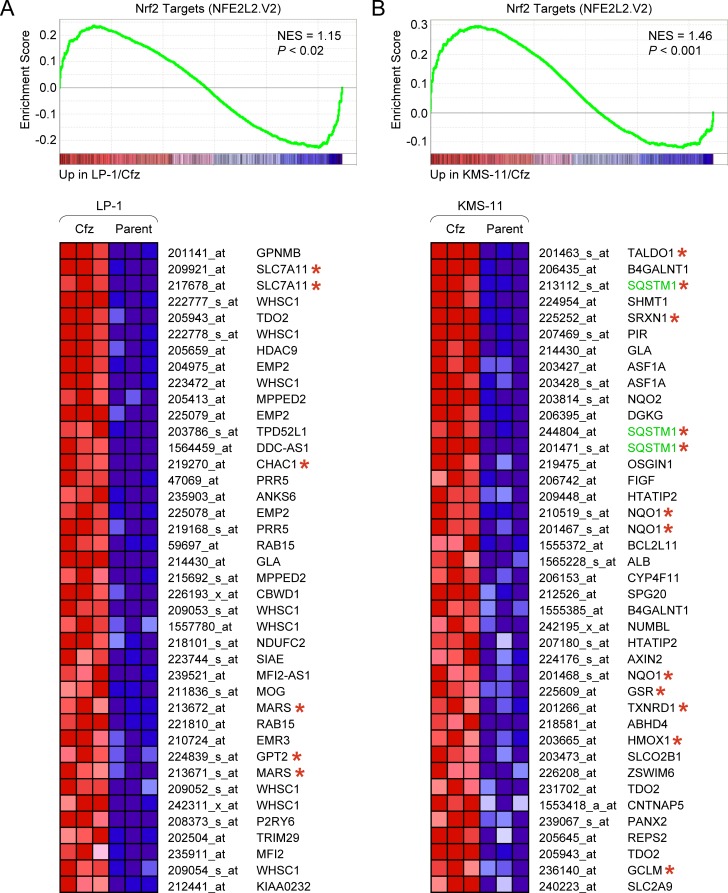
Different sets of Nrf2 target genes are upregulated in LP-1/Cfz and KMS-11/Cfz cells GSEA enrichment plot and heat map of the leading edge subset of Nrf2 target genes upregulated in LP-1/Cfz (Cfz) *versus* parental LP-1 (Parent) cells (triplicate samples). Selected Nrf2 targets that are coregulated by ATF4 are indicated with asterisks. GSEA enrichment plot and heat map of the leading edge subset of Nrf2 target genes upregulated in KMS-11/Cfz (Cfz) *versus* parental KMS-11 (Parent) cells (triplicate samples). Prototypical Nrf2 targets are indicated with asterisks. Probe sets for the *SQSTM1* gene are highlighted. Some Nrf2 targets (e.g., *TDO2* and *GLA*) were upregulated in both LP-1/Cfz and in KMS-11/Cfz cells. Gene set: NFE2L2.V2 (M2870). NES, normalized enrichment score.

We first applied GSEA to examine gene sets from the C3:TFT (transcription factor targets) subcollection of the Molecular Signatures Database (MSigDB). Binding site motifs for the “cap ‘n’ collar” transcription factors NF-E2 (V$NFE2_01) and Nrf2 (V$NRF2_Q4) that recognize a similar AP1-like core consensus sequence (TGA(G/C/T)TCA) [40] were significantly enriched in genes with increased expression in LP-1/Cfz and KMS-11/Cfz cells (Figure S2). Although NF-E2 expression is restricted to erythroid cells, Nrf2 is broadly expressed and activated in response to stress [41, 42]. In line with Nrf2 activation in both carfilzomib-resistant MM models, significant enrichment of Nrf2 target genes (NFE2L2.V2 gene set) was observed when GSEA was applied to the C6 (oncogenic signatures) collection of MSigDB (Figure 1).

GSEA detects coordinated expression changes in groups of genes by taking all genes into account, even if individual representatives of the groups exhibit modest changes [43]. In a second, complementary approach, we employed fold change (FC) cutoffs to compare similar numbers of differentially expressed genes in LP-1/Cfz (FC ≥ 1.7; 896 probe sets) and KMS-11/Cfz cells (FC ≥ 1.4; 887 probe sets). Transcription factor binding site motif discovery was carried out using the oPOSSUM-3 Single Site Analysis tool and the JASPAR CORE vertebrate database [44]. NFE2L2 was the highest-scoring motif in KMS-11/Cfz cells and the only motif in common among the top ten ranked transcription factor binding sites overrepresented in the promoter regions of the upregulated genes in both carfilzomib-resistant MM models (Table 1). This result was notable because an Nrf2 ChIP-seq dataset was one of the data sources used to validate the performance of the oPOSSUM-3 program [41, 44]. The Nrf2 ChIP-seq dataset comprised basal (*Nrf2*^−/−^) and inducible (*Keap1*^−/−^) direct binding targets of Nrf2 in mouse embryo fibroblasts [41]. We combined this dataset with Nrf2 direct binding targets identified by ChiP-seq experiments in human lymphoblastoid cells after treatment with the dietary isothiocyanate, sulforaphane [40]. Using GeneSpring analysis software, we found significant enrichment of these Nrf2 direct binding targets in the differentially expressed genes in LP-1/Cfz cells (102 out of 896 probe sets; *P* < 10^−31^) and KMS-11/Cfz cells (107 out of 887 probe sets; *P* < 10^−31^) (see Table S1 for the probe set lists). Moreover, there was also significant overlap of the differentially expressed genes in the carfilzomib-resistant MM cells and genes that were downregulated in human lymphoblastoid cells after sulforaphane treatment — LP-1/Cfz cells (72 out of 896 probe sets; *P* < 10^−20^) and KMS-11/Cfz cells (68 out of 887 probe sets; *P* < 10^−20^) — which may represent indirect Nrf2 targets (see Table S2 for the probe set lists).

**Table 1:** Overrepresented transcription factor binding site (TFBS) motifs in genes with increased expression in LP-1/Cfz and KMS-11/Cfz cells*

A. LP-1/Cfz cells				
TFBS	JASPAR ID	Class	Family	Fisher Score
Arnt::Ahr	MA0006.1	Zipper-Type	Helix-Loop-Helix	27.7
Hand1::Tcfe2a	MA0092.1	Zipper-Type	Helix-Loop-Helix	26.2
SP1	MA0079.2	Zinc-coordinating	BetaBetaAlpha-zinc finger	24.3
AP1	MA0099.2	Zipper-Type	Leucine Zipper	24.1
Nkx3-2	MA0122.1	Helix-Turn-Helix	Homeo	24.0
MEF2A	MA0052.1	Other Alpha-Helix	MADS	23.4
TBP	MA0108.2	Beta-sheet	TATA-binding	23.0
NFATC2	MA0152.1	Ig-fold	Rel	21.9
MZF1_5-13	MA0057.1	Zinc-coordinating	BetaBetaAlpha-zinc finger	21.8
NFE2L2	MA0150.1	Zipper-Type	Leucine Zipper	21.4

B. KMS-11/Cfz cells				
TFBS	JASPAR ID	Class	Family	Fisher Score
NFE2L2	MA0150.1	Zipper-Type	Leucine Zipper	22.7
NFYA	MA0060.1	Other Alpha-Helix	NFY CCAAT-binding	17.3
Spz1	MA0111.1	Other	Other	14.2
Foxa2	MA0047.2	Winged Helix-Turn-Helix	Forkhead	13.4
INSM1	MA0155.1	Zinc-coordinating	BetaBetaAlpha-zinc finger	13.2
RELA	MA0107.1	Ig-fold	Rel	13.0
REL	MA0101.1	Ig-fold	Rel	12.7
Esrrb	MA0141.1	Zinc-coordinating	Hormone-nuclear Receptor	12.5
FEV	MA0156.1	Winged Helix-Turn-Helix	Ets	12.5
CEBPA	MA0102.2	Zipper-Type	Leucine Zipper	12.4

* Identified by oPOSSUM-3 Single Site Analysis using JASPAR CORE vertebrate profiles (minimum information content = 8 bits; matrix score threshold: 85%) and ranked by Fisher score. Search regions: 5,000 bp upstream and 5,000 bp downstream of transcription start sites. The NFE2L2 binding site motif was identified in both LP-1/Cfz and KMS-11/Cfz upregulated gene sets. Fisher scores reflect the number of genes containing the predicted TFBS.

Strikingly, different Nrf2 target genes were upregulated in LP-1/Cfz and KMS-11/Cfz cells. Prototypical cytoprotective Nrf2 targets, as exemplified by *NQO1* [15], were significantly upregulated in KMS11/Cfz cells [11] but not in LP-1/Cfz cells (Figure 1; Table S1). These findings suggested differential levels of Nrf2 activation and/or that the Nrf2-interacting partners might be different in the two carfilzomib-resistant MM model systems. In support of the latter possibility, GSEA indicated activation of a prosurvival ATF4 target gene response in LP-1/Cfz cells (Figure S3A, S3B). GSEA also showed enrichment of HER2/ERBB2-related signatures in LP-1/Cfz cells when the C2:CP (canonical pathways) subcollection of MSigDB was queried (Figure S3C, S3D). Both factors were previously reported to cooperate with Nrf2 and modulate specificity of Nrf2 target gene activation [35, 36, 45, 46]. In particular, a number of the Nrf2 targets upregulated in LP-1/Cfz cells (indicated in Figure 1A) were previously shown to be directly coregulated by ATF4 [34, 47]. Regardless of the different transcriptional outcomes, taken together, the data implicated activation of Nrf2 pathways in the acquisition of carfilzomib resistance in both MM models. In the following experiments, we concentrated mainly on elucidating the molecular mechanisms and potential functional significance of Nrf2 pathway activation in LP-1/Cfz cells.

### The autophagy-related gene *GABARAPL1* is an Nrf2 target upregulated in carfilzomib-resistant MM cells

**Figure 2:**
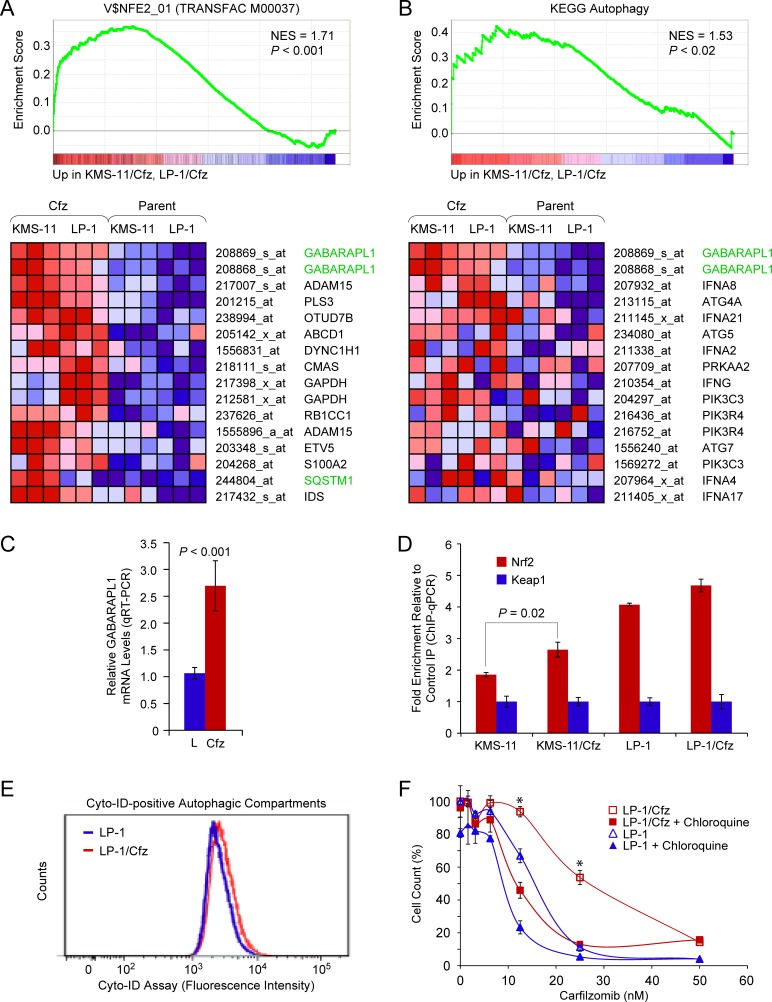
The autophagy-related gene *GABARAPL1* is an Nrf2 binding target upregulated in LP-1/Cfz and KMS-11/Cfz cells **A.** GSEA enrichment plot and heat map of the leading edge subset of genes upregulated in both LP-1/Cfz and KMS-11/Cfz cells (triplicate samples) whose promoter regions contain the NF-E2 motif. Gene set: V$NFE2_01 (M1608). The top-ranked probe sets corresponded to *GABARAPL1*. **B.** GSEA enrichment plot and heat map of the leading edge subset of autophagy pathway genes upregulated in both LP-1/Cfz and KMS-11/Cfz cells. The top-ranked probe sets corresponded to *GABARAPL1*. Gene set: KEGG Regulation of autophagy (M6382; KEGG Pathway hsa04140). qRT-PCR analysis was performed to validate the differential expression of *GABARAPL1* mRNA in LP-1/Cfz (Cfz) *versus* parental LP-1 (L) cells (mean values of three qRT-PCR experiments). See Table S1A for expression changes determined from the microarray data. **D.** Increased binding of Nrf2 to the GABARAPL1 promoter region indicated in Figure S5 in LP-1/Cfz and KMS-11/Cfz cells as determined by ChIP-qPCR. **E.** Fluorescence histograms of LP-1/Cfz and parental LP-1 cells stained with the Cyto-ID autophagy detection reagent. **F.** Cells were treated with the indicated concentrations of carfilzomib for 72 hours in the absence or presence of chloroquine (10 μM) and cell viability was determined by alamarBlue assay. *, *P* < 0.001 *vs* carfilzomib alone (*n* = 3).

We verified the microarray data for selected Nrf2 targets in LP-1/Cfz cells by real-time reverse transcription polymerase chain reaction (qRT-PCR) assay and western blot analysis (Figure S4; see also Figure 2C and Figure 9C). We noted that several of the novel Nrf2 targets are involved in translational control mechanisms; for example, *EEF1A2*, *EIF4E3, RND3*/*RhoE* (Table S1A) and *FAM129A*/*Niban* (Table S2A). *EEF1A2* is a translation elongation factor gene that was previously shown to promote survival of mouse plasmacytoma cells [48], whereas *EIF4E3*, *RND3*/*RhoE* and *FAM129A*/*Niban* encode proteins that participate in various facets of cap-dependent translation initiation (described in more detail below) [38, 49, 50].

Of the validated genes, we focused initially on the autophagy-related gene *GABARAPL1* [51] since it was also significantly upregulated in KMS-11/Cfz cells (Table S1B). Indeed, inspection of the enriched V$NFE2_01 transcription factor motif gene set indicated that *GABARAPL1* was the top-ranked upregulated Nrf2 target in common between LP-1/Cfz and KMS-11/Cfz cells (Figure 2A). In this regard, it was noteworthy that the KEGG autophagy gene set was among the significantly enriched gene sets in the MSigDB C2:CP (canonical pathways) collection identified by combined pairwise comparison of LP-1/Cfz and KMS-11/Cfz *versus* parental LP-1 and KMS-11 cells, and *GABARAPL1* was the top-ranked gene in this instance as well (Figure 2B). To investigate whether *GABARAPL1* is an Nrf2-binding target in LP-1/Cfz and KMS-11/Cfz cells, we performed ChIP-qPCR analysis on the promoter region of the *GABARAPL1* gene.

An evolutionarily conserved Nrf2 motif was identified in the GABARAPL1 promoter region by the ConTra v2 transcription factor binding site motif discovery program using position weight matrices from both the JASPAR CORE and TRANSFAC database libraries [52] (Figure S5A). Of note, this motif coincided with an NF-E2 ChIP-seq binding site identified in K562 erythroid cells by the ENCODE project (Figure S5B). Using primers flanking this site, we found specific enrichment of Nrf2-precipitated chromatin in comparison to the unrelated antibody control precipitation in both MM cell lines, increased binding of which was observed in the carfilzomib-resistant derivatives (Figure 2D). This result is congruent with previous findings of substantial overlap of functional Nrf2 and NF-E2 binding sites at the genome-wide and individual gene levels [40, 42]. Thus, the data support the notion that *GABARAPL1* is a direct Nrf2 target gene in LP-1/Cfz and KMS-11/Cfz cells.

GABARAPL1 is involved in selective autophagy through its interaction with ubiquitin-binding cargo receptors such as SQSTM1/p62 [53] and it is essential during the late stages of autophagosome maturation [54]. Previously, we showed that increased autophagic flux contributes to carfilzomib resistance in KMS-11/Cfz cells [11]. Increased staining intensity with the Cyto-ID autophagy detection reagent revealed that LP-1/Cfz cells also had higher steady state levels of autophagosomes (Figure 2E), indicating that activation of the Nrf2-*GABARAPL1* axis correlates with increased autophagic activity [55]. We also previously demonstrated that inhibition of autophagy by chloroquine treatment, which increases lysosomal pH and blocks autophagosome-lysosome fusion, sensitized KMS-11/Cfz cells to carfilzomib [11]. Cotreatment with chloroquine likewise diminished carfilzomib resistance in LP-1/Cfz cells (Figure 2F), indicating that prosurvival autophagy contributes to acquired carfilzomib resistance in this MM cell line similarly to KMS-11/Cfz cells.

### LP-1/Cfz cells exhibit increased antioxidant capacity due to altered intermediary metabolism

As mentioned above, Nrf2 targets directly involved in reactive oxygen species (ROS) detoxification were not significantly upregulated in LP-1/Cfz cells by comparison to parental LP-1 cells. However, examination of the microarray data revealed that a number of these prototypical Nrf2 target genes (including *NQO1*, *GCLC*, *GCLM*, *GPX4*, *GSR*, *GSTM1*) are already highly expressed in LP-1 cells. NQO1 ((NAD(P)H:quinone oxidoreductase 1), GSR (glutathione reductase) and other antioxidant-associated enzymes regulated by Nrf2 require NADPH as a reducing cofactor [15]. Accordingly, Nrf2 also regulates several NADPH-generating enzymes. Specifically, Nrf2 facilitates NADPH production by directing carbon flux through the pentose phosphate pathway [56]. Consistent with the hypothesis that Nrf2 is activated in LP-1/Cfz as well as in KMS-11/Cfz cells, NADPH levels were increased concomitant with upregulation of KEGG pentose phosphate pathway genes in both models (Figure S6).

**Figure 3:**
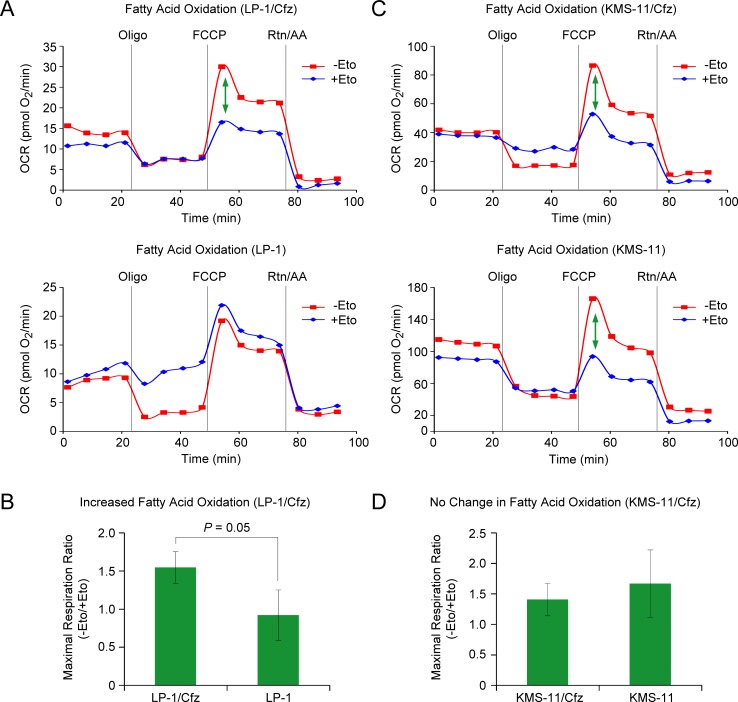
LP-1/Cfz cells exhibit increased FAO **A.** Oxygen consumption rate (OCR) was measured using the Seahorse XF24 Extracellular Flux Analyzer. FAO was determined by XF Cell Mito Stress Test using the XF Palmitate-BSA FAO substrate in the absence or presence of etomoxir (Eto), an inhibitor of carnitine palmitoyltransferase-1, the rate limiting enzyme in FAO. The ATP synthase inhibitor oligomycin (Oligo), the uncoupler carbonyl cyanide 4-(trifluoromethoxy) phenylhydrazone (FCCP), and the complex I and III inhibitors rotenone and antimycin A (Rtn/AA) were injected at the indicated times. The green arrow indicates the difference in maximal respiration — due to FAO — when LP-1/Cfz cells were treated with FCCP in the absence or presence of Eto. **B.** Average increase in FAO in LP-1/Cfz *versus* parental LP-1 cells (*n* = 3). Oxidation of fatty acids was determined in KMS-11/Cfz and KMS-11 cells as described in A. No change in FAO in KMS-11/Cfz *versus* parental KMS-11 cells (*n* = 3).

It is becoming increasingly appreciated that Nrf2 also affects multiple aspects of intermediary metabolism indirectly [15], including mitochondrial fatty acid oxidation (FAO) [57]. Of note, during the metabolic shift towards FAO, activation of autophagy hydrolyzes lipids into fatty acids for fuel [58]. Considering that FAO is an important source of NADPH in leukemic cells [59], we examined whether rates of FAO were increased in carfilzomib-resistant LP-1/Cfz and KMS-11/Cfz cells. Increased FAO was observed in LP-1/Cfz *versus* parental LP-1 cells (Figure 3A, 3B). By comparison, basal rates of FAO were already elevated in parental KMS-11 cells and there was no further increase in KMS-11/Cfz cells (Figure 3C, 3D).

**Figure 4:**
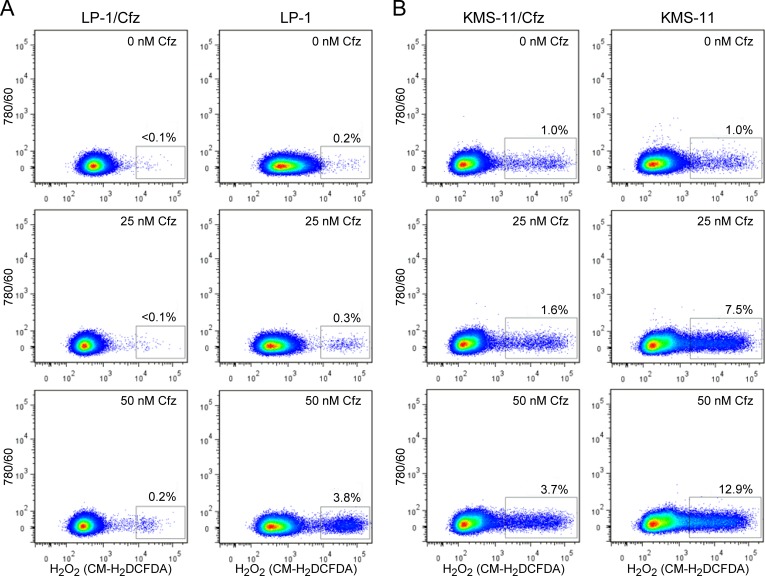
LP-1/Cfz and KMS-11/Cfz cells contain lower levels of hydrogen peroxide-associated ROS than parental LP-1 and KMS-11 cells following carfilzomib treatment **A.** LP-1/Cfz and parental LP-1 cells were treated for 18 hours with the indicated concentrations of carfilzomib (Cfz) and cellular hydrogen peroxide (H_2_O_2_) levels were quantified by flow cytometry using the redox-sensitive CM-H_2_DCFDA fluorescent dye. **B.** KMS-11/Cfz and parental KMS-11 cells were treated for 18 hours with the indicated concentrations of carfilzomib and cellular hydrogen peroxide (H_2_O_2_) levels were quantified by flow cytometry using the redox-sensitive CM-H_2_DCFDA fluorescent dye.

**Figure 5:**
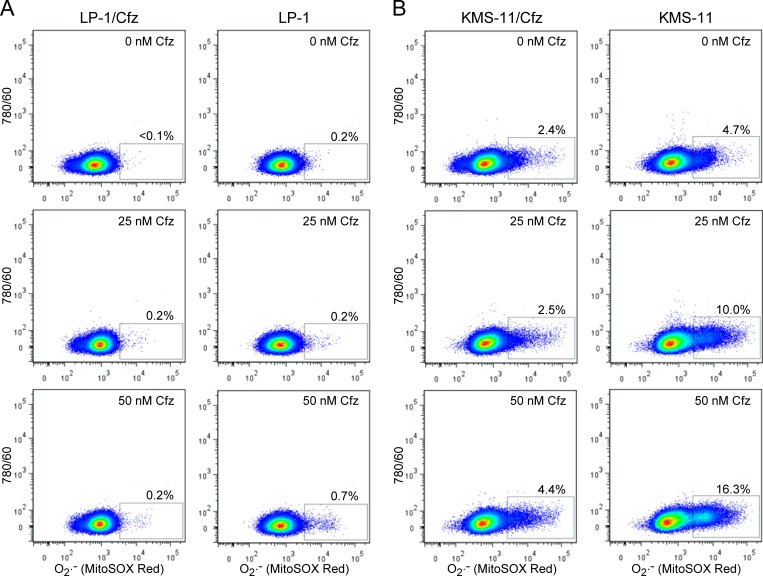
LP-1/Cfz and KMS-11/Cfz cells contain lower levels of superoxide than parental LP-1 and KMS-11 cells following carfilzomib treatment **A.** LP-1/Cfz and parental LP-1 cells were treated for 18 hours with the indicated concentrations of carfilzomib (Cfz) and superoxide (O_2_^.−^) levels were quantified by flow cytometry using the redox-sensitive MitoSOX Red fluorescent dye. **B.** KMS-11/Cfz and parental KMS-11 cells were treated for 18 hours with the indicated concentrations of carfilzomib and superoxide (O_2_^.−^) levels were quantified by flow cytometry using the redox-sensitive MitoSOX Red fluorescent dye.

Considered together, the gene expression profiling and functional assays supporting increased NADPH production and/or upregulation of genes that detoxify ROS suggested that LP-1/Cfz and KMS-11/Cfz cells would exhibit enhanced Nrf2-mediated antioxidant capacity. To examine this, we measured ROS generation using the fluorescent redox-sensitive dyes CM-H_2_DCFDA, which is specific for hydrogen peroxide, and MitoSOX Red, which is selective for superoxide [60]. Cells were treated with varying concentrations of carfilzomib and ROS levels were quantified by flow cytometry. Lower levels of ROS were generated in LP-1 cells compared to KMS-11 cells under all conditions; at the highest carfilzomib concentrations, both hydrogen peroxide (Figure 4) and superoxide (Figure 5) levels were reduced by more than 70% in the carfilzomib-resistant derivatives *versus* their parental counterparts.

**Figure 6:**
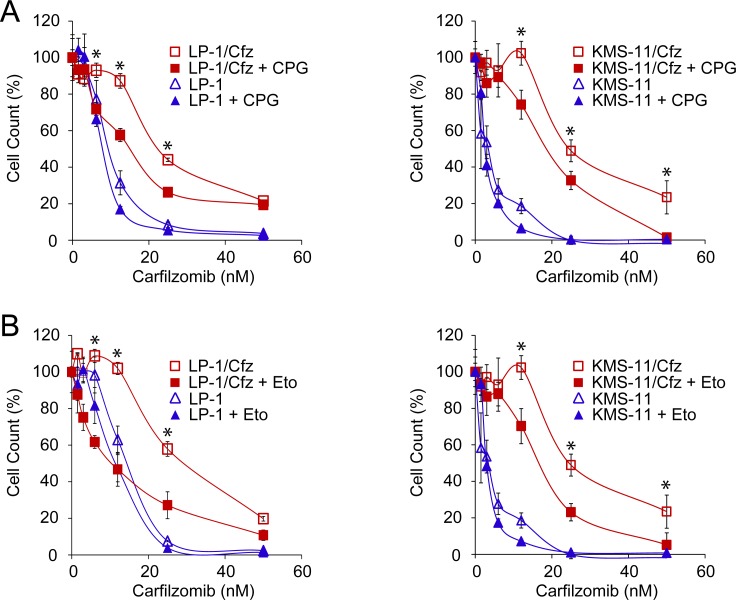
Inhibition of glutathione homeostasis or FAO sensitizes LP-1/Cfz and KMS-11/Cfz cells to carfilzomib **A.** Treatment with (S)-4-carboxyphenylglycine (CPG), an inhibitor of xCT (a subunit of the x_c_^−^ cystine antiporter involved in glutathione homeostasis encoded by the *SLC7A11* gene) sensitizes LP-1/Cfz cells (left graph) and KMS-11/Cfz cells (right graph) to carfilzomib. Cells were treated with the indicated concentrations of carfilzomib for 72 hours in the absence or presence of CPG (0.2 mM) and cell viability was determined by alamarBlue assay. *, *P* < 0.001 *vs* carfilzomib alone (*n* = 3). **B.** Treatment with etomoxir, an inhibitor of carnitine palmitoyltransferase-1 (the rate limiting enzyme in FAO), sensitizes LP-1/Cfz cells (left graph) and KMS-11/Cfz cells (right graph) to carfilzomib. Cells were treated with the indicated concentrations of carfilzomib for 72 hours in the absence or presence of etomoxir (20 μM) and cell viability was determined by alamarBlue assay. *, *P* < 0.001 *vs* carfilzomib alone (*n* = 3).

To determine whether antioxidant defense conferred protection to carfilzomib in these MM models and whether there was a contribution of FAO, the cells were cotreated with carfilzomib and either (S)-4-carboxyphenylglycine, an inhibitor of xCT (a subunit of the x_c_^−^ cystine antiporter involved in glutathione homeostasis encoded by the *SLC7A11* gene; see Figure 1A) [34] or etomoxir, an inhibitor of carnitine palmitoyltransferase-1 (the rate limiting enzyme in FAO; see Figure 3) [61]. Cotreatment with either inhibitor resulted in enhanced sensitivity of the resistant cells to carfilzomib (Figure 6), indicating that the glutathione-based antioxidant system contributes to carfilzomib resistance in these MM models and that FAO is a potential source of reducing equivalents underlying the increased ROS defense.

**Figure 7:**
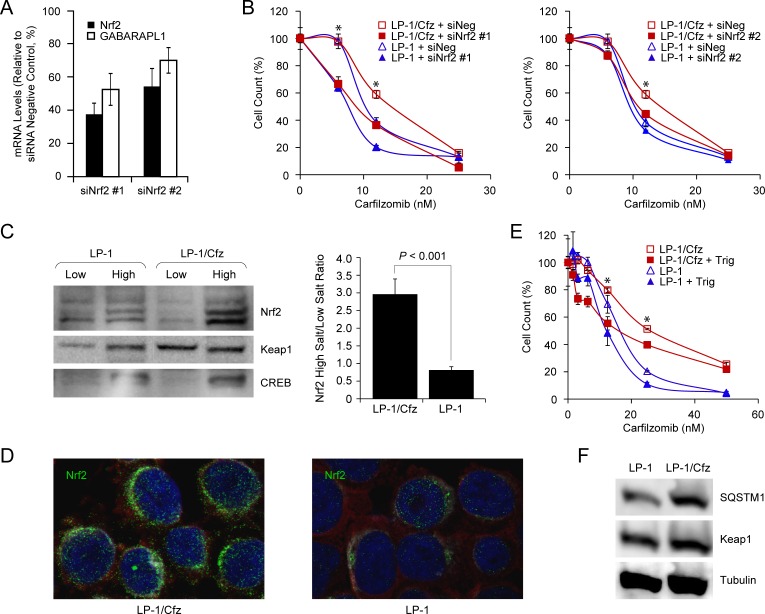
Nrf2 activation in LP-1/Cfz cells confers resistance to carfilzomib **A.** Knockdown of Nrf2 mRNA using two specific siRNAs (siNrf2 #1, siNrf2 #2) was accompanied by downregulation of GABARAPL1 mRNA (mean values of three qRT-PCR experiments). **B.** Knockdown of Nrf2 mRNA using two specific siRNAs (siNrf2 #1, siNrf2 #2) sensitizes LP-1/Cfz cells to carfilzomib. Cells were treated with the indicated concentrations of carfilzomib for 48 hours after transient transfection and cell viability was determined by alamarBlue assay. *, *P* < 0.001 *vs* negative siRNA control (siNeg, *n* = 3). **C.** Western blot analysis demonstrating significantly higher Nrf2 levels in the high salt fraction of LP-1/Cfz cell lysates (*P* < 0.001; *n* = 4). **D.** Cells were labeled with anti-Nrf2 antibody (Alexa Fluor 488, green) and immunofluorescence staining was analyzed by confocal laser scanning microscopy. **E.** Cells were treated with the indicated concentrations of carfilzomib for 48 hours in the absence or presence of trigonelline (Trig, 1 μM), an inhibitor of Nrf2 nuclear import, and cell viability was determined by alamarBlue assay. *, *P* < 0.001 *vs* carfilzomib alone (*n* = 3). **F.** Western blot analysis showing increased SQSTM1 levels in LP-1/Cfz compared to parental LP-1 cells (*P* < 0.03; *n* = 5).

Finally, to directly test whether Nrf2 activity contributes to carfilzomib resistance, we transfected LP-1/Cfz and parental LP-1 cells with two previously validated Nrf2 siRNAs and a control siRNA with no known mammalian homology [62, 63]. We confirmed that Nrf2 mRNA was knocked down ~45-65% by qRT-PCR. This was accompanied by an ~30-45% decrease in GABARAPL1 mRNA levels (Figure 7A). As illustrated in Figure 7B, both Nrf2 siRNAs sensitized LP-1/Cfz cells to carfilzomib.

### Activation of the PERK-eIF2α axis contributes to SQSTM1/p62 synthesis and carfilzomib resistance in LP-1/Cfz cells

Next, we focused on the molecular mechanisms underlying Nrf2 activation in LP-1/Cfz cells. Nrf2 mRNA levels were not significantly increased. However, western blot analyses showed significantly higher Nrf2 protein levels in the high salt (nuclear) fraction of LP-1/Cfz cell lysates compared to parental LP-1 cell lysates (*P* < 0.001) (Figure 7C). Immunofluorescence confocal microscopy confirmed that Nrf2 had a predominantly perinuclear and nuclear localization in LP-1/Cfz cells (Figure 7D) [64]. Moreover, treatment with trigonelline, an inhibitor of Nrf2 nuclear import [63], sensitized LP-1/Cfz cells to carfilzomib (Figure 7E). Steady state Keap1 levels were not reduced in LP-1/Cfz cells compared to parental LP-1 cells (Figure 7F). Unexpectedly, however, despite the fact that SQSTM1 mRNA levels were not increased, SQSTM1/p62 protein levels were higher in LP-1/Cfz cells (Figure 7F).

Kampmann and colleagues recently reported that knockdown of the 19S proteasome subunit genes desensitized U266 MM cells to carfilzomib [65]. Because the 19S regulator delivers substrates to the 20S catalytic core, they hypothesized that a loss in 19S function may lead to the selective accumulation of certain proteins. SQSTM1/p62 was one of the proteins that accumulated upon knockdown [65]. Congruent with their results, GSEA indicated that many of the proteasome 19S subunit genes were downregulated in LP-1/Cfz cells (Figure S7). Therefore, it is possible that this mechanism may contribute to increased SQSTM1/p62 levels in LP-1/Cfz cells. However, as described below, we found that elevated protein synthesis was a contributing factor to the observed differences in SQSTM1/p62 levels. In this context, it is worth mentioning that knockdown of the 19S proteasome subunit genes in U266 MM cells was not associated with substantially increased 20S chymotrypsin-like protease activity selectively inhibited by carfilzomib [65]. Similarly, we did not detect a significant increase in 20S chymotrypsin-like protease activity in LP-1/Cfz cells (data not shown), which was consistent with only a slight (~1.3-fold) increase in mRNA of the *PSMB5*-encoded constitutive β5 subunit targeted by carfilzomib (Figure S7).

**Figure 8:**
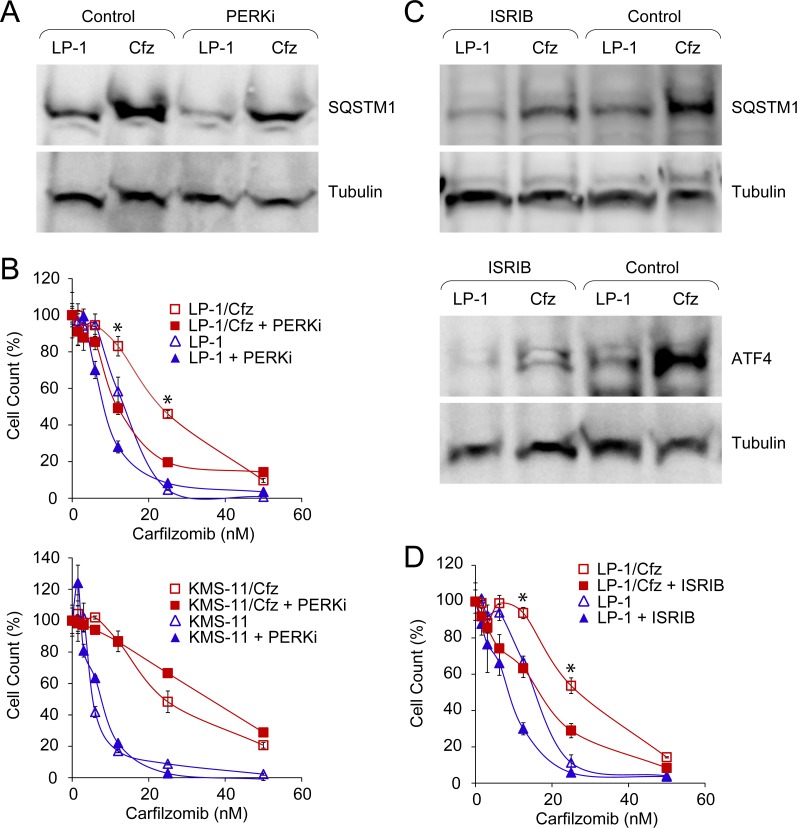
Constitutive PERK-eIF2α signaling is associated with carfilzomib resistance in LP-1/Cfz cells **A.** Western blot analysis showing that treatment with a PERK inhibitor (PERKi) lowers SQSTM1 levels in LP-1/Cfz (Cfz) and parental LP-1 cells (*P* < 0.05; *n* = 8). Cells were treated with MG-132 (15 μM) for 18 hours in the absence or presence of GSK2656157 (10 μM). **B.** Inhibition of PERK sensitizes LP-1/Cfz (top graph) but not KMS-11/Cfz cells (bottom graph) to carfilzomib. Cells were treated with the indicated concentrations of carfilzomib for 72 hours in the absence or presence of GSK2656157 (PERKi, 10 μM) and cell viability was determined by alamarBlue assay. *, *P* < 0.001 *vs* carfilzomib alone (*n* = 3). **C.** Western blot analysis showing that ISRIB treatment decreases SQSTM1 levels (*P* < 0.03; *n* = 3) (top panels) and ATF4 levels (bottom panels) in LP-1/Cfz and parental LP-1 cells. Cells were treated with MG-132 (15 μM) for 18 hours in the absence or presence of ISRIB (20 nM). **D.** Inhibition of eIF2α sensitizes LP-1/Cfz cells to carfilzomib. Cells were treated with the indicated concentrations of carfilzomib for 48 hours in the absence or presence of ISRIB (20 nM) and cell viability was determined by alamarBlue assay. *, *P* < 0.001 *vs* carfilzomib alone (*n* = 3).

Kampmann and colleagues also identified genes involved in other nodes of the proteostasis network that desensitized U266 MM cells to carfilzomib, including components of the eIF4F translation initiation complex (for example, *EIF4E1*) and the mechanistic target of rapamycin (*MTOR*) [65]. GSEA indicated that both of these pathways were downregulated in LP-1/Cfz cells: BIOCARTA_EIF4_PATHWAY (*P* < 0.001) (Figure S8A) and HALLMARK_ MTORC1_SIGNALING (*P* < 0.009) (Figure S8B). Since activation of the PERK-ATF4 pathway was also implicated by GSEA (Figure S3A) and PERK inhibits mTORC1 during induction of prosurvival autophagy [66], we assessed potential involvement of the PERK-eIF2α axis on SQSTM1/p62 translation. For these studies, we tested a highly selective inhibitor of PERK kinase activity (GSK2656157) [67] or another small molecule (ISRIB) which acts downstream and reverses the effects of phosphorylated eIF2α, thereby blocking ATF4 synthesis [68]. We found that PERK inhibition significantly reduced SQSTM1/p62 levels in LP-1/Cfz cells (Figure 8A) and sensitized LP-1/Cfz cells to carfilzomib (Figure 8B, top graph). By comparison, KMS-11/Cfz cells were not sensitized by PERK inhibition (Figure 8B, bottom graph). Treatment of LP-1/Cfz cells with ISRIB also decreased SQSTM1/p62 levels (Figure 8C, top panels) (*P* < 0.03; *n* = 3) while concomitantly diminishing ATF4 levels (Figure 8C, bottom panels). Further, ISRIB treatment sensitized LP-1/Cfz cells to carfilzomib (Figure 8D). Thus, these results indicated that activation of the PERK-eIF2α pathway contributed to carfilzomib resistance. Moreover, they argued that SQSTM1/p62 translation occurs in part *via* an ATF4-like uORF-mediated mechanism (see Table S2 of ref. [69] for details of SQSTM1 uORF-containing transcripts).

Interestingly, we did not observe enrichment of IRE1 or ATF6 expression signatures corresponding to the other branches of the UPR in LP-1/Cfz cells. In fact, GSEA indicated that UPR signaling was attenuated (HALLMARK_UNFOLDED_PROTEIN_RESPONSE downregulated; Figure S8C). Therefore, the mechanistic basis for PERK activation in LP-1/Cfz cells is unknown. However, alternative signals for PERK activation have recently been uncovered [28, 29, 66, 70]. In particular, Gupta and colleagues described a noncanonical mechanism of PERK-Nrf2 activation in the absence of an endoplasmic reticulum stress response that results from an epithelial-to-mesenchymal transition (EMT) [28, 70]. Along these lines, enrichment of an EMT-like expression signature (HALLMARK_EPITHELIAL_MESENCHYMAL_TRANSITION) was indicated by GSEA in LP-1/Cfz cells (Figure S8D), and decreased cell surface expression of E-cadherin compared to parental LP-1 cells (Figure S8E) is consistent with this as a contributory mechanism [71, 72].

### Induction of Nrf2 target genes in LP-1/Cfz cells creates a positive feedback loop promoting eIF4E3-driven Nrf2 translation

**Figure 9:**
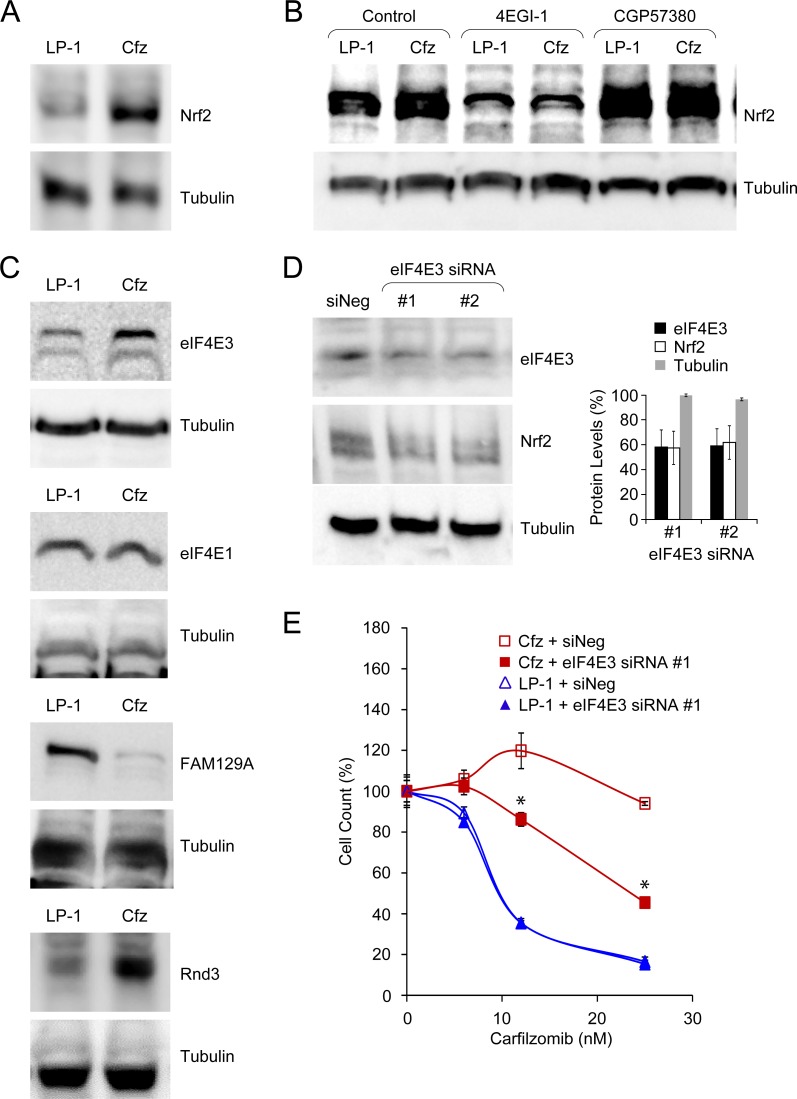
Acquisition of carfilzomib resistance in LP-1/Cfz cells is associated with eIF4E3-mediated translational reprogramming **A.** Western blot analysis showing increased Nrf2 levels in LP-1/Cfz (Cfz) *versus* parental LP-1 cells (*P* < 0.002; *n* = 18). Cells were treated with MG-132 (15 μM) for 18 hours. **B.** Synthesis of Nrf2 depends on eIF4E/eIF4G interaction (*P* < 0.003; *n* = 10) and is enhanced upon MNK inhibition (*P* < 0.03; *n* = 6). Cells were treated with MG-132 (15 μM) for 18 hours in the absence or presence of an eIF4E/eIF4G interaction inhibitor (4EGI-1, 50 μM) or an MNK inhibitor (CGP57380, 10 μM) and whole cell lysates were analyzed by Western blotting. **C.** Western blot analysis showing (from top to bottom) increased eIF4E3 levels (*P* < 0.001; *n* = 4), similar eIF4E1 levels, decreased FAM129A levels (*P* < 1 × 10^−4^; *n* = 7), and increased Rnd3 levels (*P* < 0.02; *n* = 6) in LP-1/Cfz *versus* parental LP-1 cells. Cells were treated with MG-132 (15 μM) for 18 hours. **D.** Western blot analysis showing that knockdown of EIF4E3 mRNA using two specific siRNAs was accompanied by decreased eIF4E3 and Nrf2 protein levels. **E.** Knockdown of EIF4E3 mRNA (siRNA #1) sensitizes LP-1/Cfz cells to carfilzomib. Cells were treated with the indicated concentrations of carfilzomib for 48 hours after transient transfection and cell viability was determined by alamarBlue assay. *, *P* < 0.001 *vs* negative siRNA control (siNeg, *n* = 3).

Although Nrf2 levels are primarily governed by interaction with Keap1, accumulating evidence has highlighted the importance of *de novo* translation [22–24]. Therefore, we were prompted to compare Nrf2 synthesis in LP-1/Cfz and parental LP-1 cells. For these analyses, the cells were pretreated with MG-132 to prevent Nrf2 proteasomal degradation. Under these conditions, we found significantly higher levels of Nrf2 in LP-1/Cfz cells (Figure 9A). We next tested the effects of 4EGI-1, an inhibitor of cap-dependent translation that prevents binding of eukaryotic initiation factor 4G (eIF4G) to eIF4E family members (the best characterized of which is eIF4E1) within the eIF4F translation initiation complex [73]. Inhibition of the eIF4E/eIF4G interaction eliminated the differences in Nrf2 levels between LP-1/Cfz and parental LP-1 cells (Figure 9B). The data thus indicated more active cap-dependent synthesis of Nrf2 in LP-1/Cfz cells. This result was contrary to the GSEA prediction that canonical eIF4F-mediated translation initiation was downregulated in LP-1/Cfz compared to parental LP-1 cells (Figure S8A) and suggested the possible involvement of an alternative eIF4F cap-binding complex containing another member of the eIF4E family [74]. Along these lines, it has recently been appreciated that eIF4E3 interacts with eIF4G and competes with eIF4E1 to form a novel eIF4F cap-binding complex [37, 38]. Because this Nrf2 target was upregulated at the mRNA level in LP-1/Cfz cells (Table S1A), we were interested in whether this was reflected at the level of eIF4E3 protein. We found that the increased *EIF4E3* mRNA levels in LP-1/Cfz cells were accompanied by elevated eIF4E3 protein levels, whereas LP-1/Cfz and parental LP-1 cells had similar levels of eIF4E1 (Figure 9C).

Phosphorylation of eIF4E1 by mitogen-activated protein kinase interacting kinases (MNK) 1 and 2 stimulates translation of a subset of mRNAs encoding proteins involved in cancer development and progression [75]. Gartenhaus and colleagues found that treatment of diffuse large B-cell lymphoma cells with an MNK inhibitor, CGP57380, reduced eIF4E1-driven translation and resulted in a compensatory increase in eIF4E3-driven translation [38]. To determine whether Nrf2 translation might be regulated *via* eIF4E1-eIF4E3 interplay, we investigated the effect of MNK inhibition on Nrf2 protein levels in LP-1/Cfz and parental LP-1 cells. We found that after CGP57380 treatment Nrf2 levels increased in both cases, with the levels in parental LP-1 cells approaching those in LP-1/Cfz cells (Figure 9B). These findings supported a role of eIF4E3 in the elevated synthesis of Nrf2 and the carfilzomib-resistant phenotype of LP-1/Cfz cells. To directly test this, we transfected LP-1/Cfz and parental LP-1 cells with two specific EIF4E3 siRNAs and a negative control siRNA. Figure 9D shows that Nrf2 protein levels decreased with eIF4E3 protein knockdown. Moreover, eIF4E3 knockdown sensitized LP-1/Cfz cells to carfilzomib (Figure 9E).

We also examined whether the protein levels of two other novel Nrf2 targets involved in the control of cap-dependent translation initiation — RND3/RhoE and FAM129A/Niban — corresponded with the differential mRNA levels observed in LP-1/Cfz and LP-1 cells (Figure S4). FAM129A inhibits PERK-mediated phosphorylation of eIF2α [50]. Additionally, FAM129A and Rnd3 reciprocally modulate the activity of 4E-BP1, an inhibitor of eIF4E1 [49, 50]. Briefly, the dephosphorylated form of 4E-BP1 binds to eIF4E1, preventing binding of eIF4G and formation of the eIF4F complex. mTORC1 phosphorylates 4E-BP1, leading to its dissociation from eIF4E1 which allows eIF4F formation and translation initiation [75]. FAM129A positively affects mTORC1 phosphorylation of 4E-BP1, thereby facilitating eIF4E1-driven translation [50]. In contrast, Rnd3 inhibits mTORC1 phosphorylation of 4E-BP1 which prevents 4E-BP1 disassociation from eIF4E1 and therefore inhibits eIF4E1 function [49]. This mechanism only pertains to eIF4E1 since 4E-BP1 does not bind to eIF4E3 [74]. FAM129A protein levels were decreased in LP-1/Cfz cells corresponding with its diminished mRNA expression. Conversely, Rnd3 protein levels were increased in LP-1/Cfz cells concomitant with increased mRNA expression (Figure 9C). Collectively, these results are consistent with a potential positive feedback process associated with acquisition of carfilzomib resistance in LP-1/Cfz cells wherein initial activation of Nrf2 leads to direct and indirect regulation of target genes — including transcriptional upregulation of *EIF4E3* — that facilitate enhanced eIF4E3-driven translation of Nrf2.

### Increased *EIF4E3* expression predicts Nrf2 target gene activation in minimal residual disease and relapsed MM patient samples

**Figure 10:**
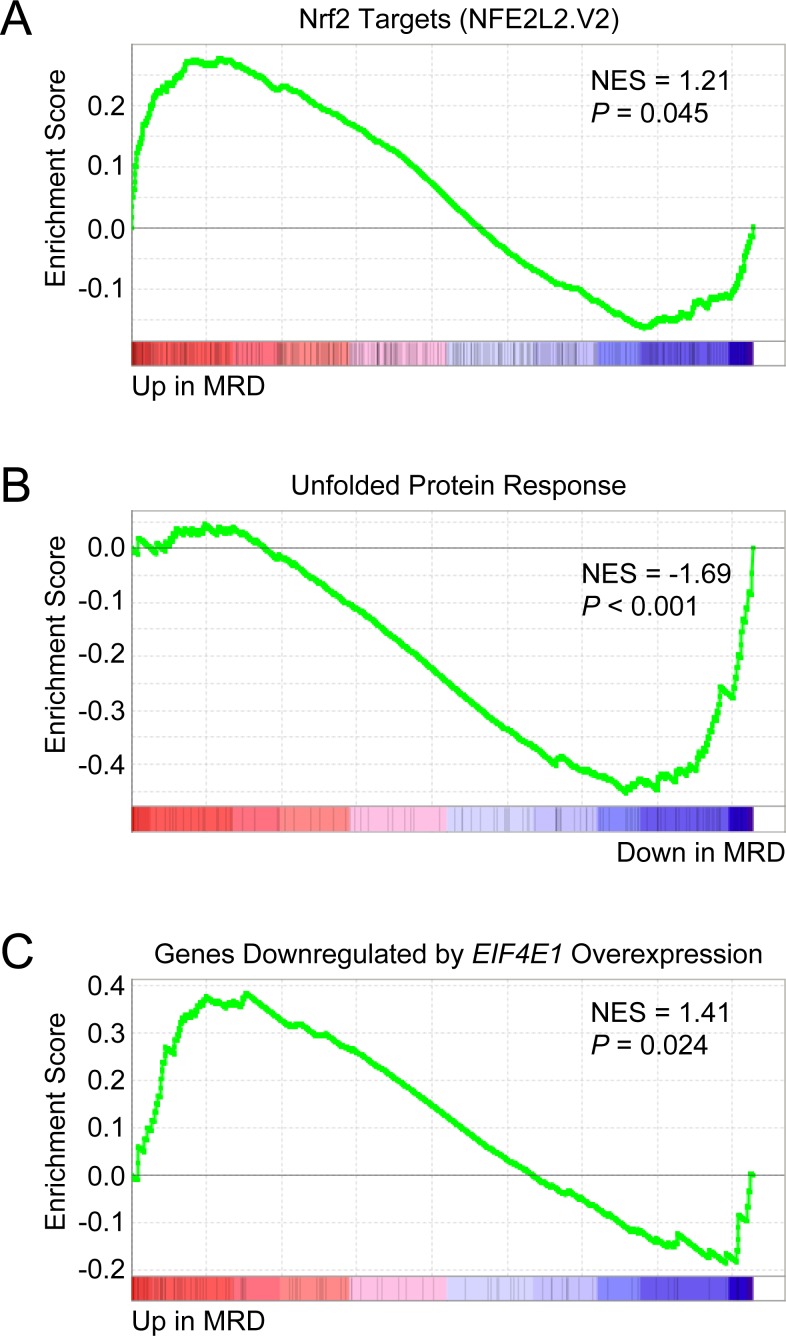
Chemoresistant minimal residual disease (MRD) cells from MM patients have increased *EIF4E3* expression and Nrf2 target gene signature enrichment Microarray data was obtained from the Gene Expression Omnibus database (www.ncbi.nlm.nih.gov/geo/; GEO accession number GSE70399) for matched MRD and diagnostic plasma cells from MM patients enrolled in the GEM2010MAS65 trial (ClinicalTrials.gov Identifier: NCT01237249). *EIF4E3* expression in MRD cells *versus* baseline was analyzed with the GEO2R web tool (http://www.ncbi.nlm.nih.gov/geo/geo2r/) and was found to be increased in 5 out of 7 samples (average fold change = 1.25; *P* = 0.013). **A.** GSEA indicated that Nrf2 target genes were upregulated in MRD cells. Gene set: NFE2L2.V2 (M2870). **B.** GSEA indicated that genes upregulated during the unfolded protein response were downregulated in MRD cells. Gene set: HALLMARK_UNFOLDED_PROTEIN_RESPONSE (M5922). **C.** GSEA indicated that genes downregulated in primary human mammary epithelial cells upon overexpression of *EIF4E1* were upregulated in MRD cells. Gene set: EIF4E_DN (M2790).

To assess the clinical relevance of our findings, we analyzed three independent publicly available gene expression datasets of MM patients. We first examined the gene expression profiles of chemoresistant minimal residual disease (MRD) and matched diagnostic samples from MM patients included in the GEM2010MAS65 clinical trial (GEO accession number GSE70399) [76]. Notably, *EIF4E3* expression was found to be increased in 5 out of 7 MRD samples (FC = 1.25; *P* = 0.013). When GSEA was applied to these 5 cases, significant upregulation of Nrf2 target genes (NFE2L2.V2 gene set) was observed in the persisting MRD cells (Figure 10A). As was found for LP-1/Cfz cells, UPR signaling was attenuated (HALLMARK_UNFOLDED_PROTEIN_ RESPONSE downregulated; Figure 10B). Moreover, GSEA predicted an inverse relationship with an enrichment pattern corresponding to genes regulated by *EIF4E1* overexpression (Figure 10C).

**Figure 11:**
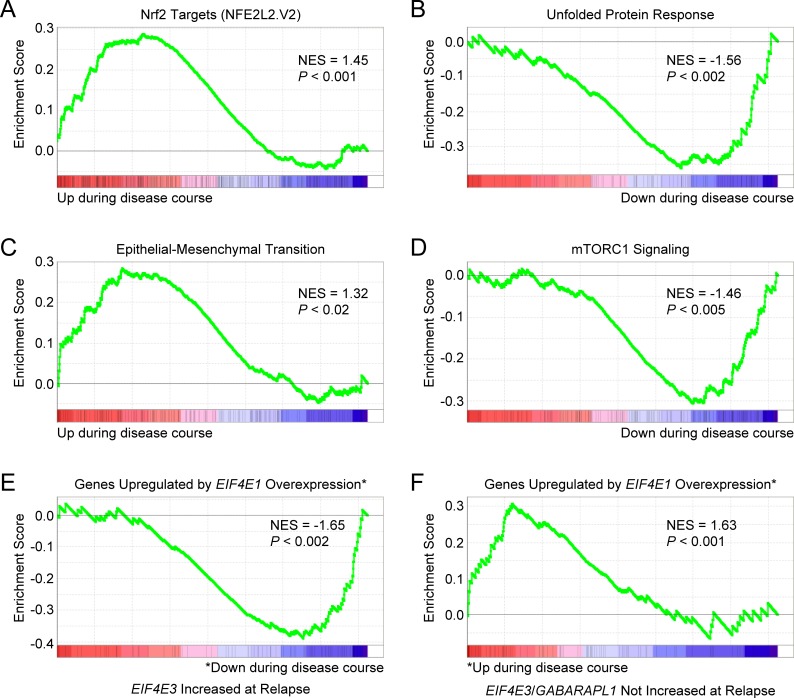
Gene expression signatures of MM cells associated with increased *EIF4E3* expression during progression of disease Microarray data was obtained from the GEO database (GEO accession number GSE36824) for patient-paired relapse and diagnostic samples from 17 patients treated with various regimens. *EIF4E3* expression was increased in 4 out of 17 cases. **A.** GSEA indicated that Nrf2 target genes were upregulated during disease course in these cases. Gene set: NFE2L2.V2 (M2870). **B.** GSEA indicated that genes upregulated during the UPR were downregulated. Gene set: HALLMARK_UNFOLDED_PROTEIN_RESPONSE (M5922). **C.** These patients had enrichment of an EMT-like expression signature. Gene set: HALLMARK_EPITHELIAL_MESENCHYMAL_ TRANSITION (M5930). **D.** Genes upregulated through activation of the mTORC1 complex were also downregulated in these cases. Gene set: HALLMARK_MTORC1_SIGNALING (M5924). **E.** GSEA indicated that genes upregulated in primary human mammary epithelial cells upon overexpression of *EIF4E1* were downregulated during disease course in relapse cells with increased *EIF4E3* expression. Gene set: EIF4E_UP (M2791). **F.** GSEA of patient-paired relapse and diagnostic samples without increased *EIF4E3* and/or *GABARAPL1* expression (10 out of 17 cases) showed enrichment of the EIF4E_UP signature. See also Figure S9.

We also examined patient-paired relapse and diagnostic samples from 17 MM patients treated with various regimens (GEO accession number GSE36824) [77]. *EIF4E3* expression was increased in 4 out of 17 cases of disease progression. GSEA indicated that Nrf2 target genes (NFE2L2.V2 gene set) were upregulated during disease course in these 4 cases (Figure 11A). As for MRD cells with increased *EIF4E3* expression, GSEA predicted lack of UPR stress in the relapsed MM cells (HALLMARK_UNFOLDED_PROTEIN_ RESPONSE downregulated; Figure 11B). These relapsed MM cells also had enrichment of an EMT-like expression signature (HALLMARK_EPITHELIAL_MESENCHYMAL_ TRANSITION; Figure 11C). Moreover, genes upregulated through activation of the mTORC1 complex were downregulated in these 4 cases (HALLMARK_MTORC1_SIGNALING; Figure 11D). In addition, GSEA again predicted an inverse correlation with an *EIF4E1*-specific gene expression signature (Figure 11E). Of the 17 samples, *GABARAPL1* expression was increased in 5 cases during disease progression, two of which also had increased *EIF4E3* expression. Performing GSEA on the combined *EIF4E3* plus *GABARAPL1* dataset (7 out of 17 cases) indicated activation of Nrf2 signaling and yielded the same enrichment patterns for the other pathways identified for the 4 cases with increased *EIF4E3* expression (data not shown).

On the other hand, when GSEA was performed on the 10 relapsed MM patient samples that did not exhibit increased *EIF4E3* or *GABARAPL1* expression, the exact opposite patterns of enrichment were obtained. In these cases, a positive correlation with *EIF4E1*-upregulated genes was observed during disease progression (Figure 11F) concomitant with a prediction of increased mTORC1 signaling (Figure S9A). Although a UPR was predicted (Figure S9B), Nrf2 target genes were downregulated (Figure S9C) and a reverse EMT-like expression signature (i.e., mesenchymal-to-epithelial transition) was suggested (Figure S9D). Therefore, these parameters appeared to separate the relapsed MM samples into two distinct groups: one, “LP-1/Cfz-like”, characterized by increased *EIF4E3* (and/or *GABARAPL1*) expression having activation of the Nrf2 signaling pathway, and a second group (“eIF4E1-like”) exhibiting the opposite phenotype and pathway activation states in which enhanced eIF4E1-driven translation was implied.

### Elevated expression of *EIF4E3* is prognostic of poor survival

**Figure 12:**
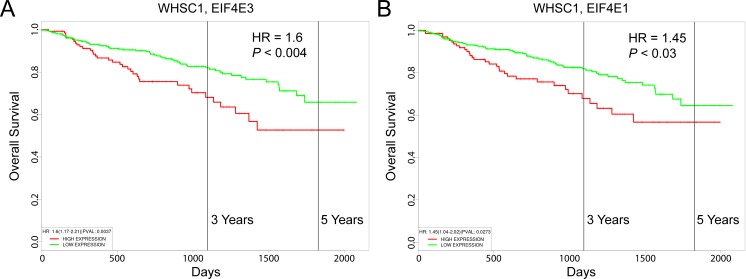
Prognostic value of *EIF4E3* expression in MM patient survival outcomes Kaplan-Meier survival plots of 546 newly diagnosed patients with MM consisting of 351 cases enrolled on total therapy 2 (TT2) and 195 patients enrolled in total therapy 3 (TT3) which incorporates bortezomib into the induction phase of the TT2 transplant regimen (GEO accession number GSE2658) were created using PROGgeneV2. **A.** Coexpression of *WHSC1* and *EIF4E3*. **B.** Coexpression of *WHSC1* and *EIF4E1*. Cohorts were divided at the 75^th^ percentile of mean gene expression. HR, hazard ratio determined by Cox proportional hazards model.

LP-1 MM cells contain the t(4;14) chromosomal translocation that activates a histone methyltransferase encoded by the *WHSC1* gene (also known as *MMSET*) [78], which is consistently associated with poor outcome [79]. Using the UAMS-70 prognostic gene signature, Shaughnessy and colleagues separated *WHSC1*-positive MM patients into higher-risk and lower-risk subgroups [80]. We were curious to know whether increased expression of *EIF4E3* was prognostic for decreased overall survival of *WHSC1*-positive MM patients. Using the PROGgeneV2 prognostic biomarker identification tool [81] as previously described [11], we performed a Cox proportional hazards regression analysis on 546 newly diagnosed MM patients treated on the UARK 98-026 TT2 and UARK 2003-033 TT3 clinical trials [80]. The hazard ratio (HR) for patients with high *versus* low *WHSC1* expression was 1.3 (95% confidence interval (CI), 1.1-1.54; *P* < 0.002). As shown in Figure 12A, high-level coexpression of *EIF4E3* resulted in increased risk of mortality (HR = 1.6; 95% CI, 1.17-2.21; *P* < 0.004), which was higher than the increased risk score predicted by high-level coexpression of *EIF4E1* (HR = 1.45; 95% CI, 1.04-2.02; *P* < 0.03) (Figure 12B).

## DISCUSSION

Despite improved outcomes with the introduction of novel agents such as proteasome inhibitors to MM treatment regimens over the past decade, patients inevitably succumb to their disease because the MM cells become resistant to the drugs [1]. MM cells can develop resistance to proteasome inhibitors through a variety of mechanisms [4]. However, it is still unclear which mechanisms explain clinical proteasome inhibitor resistance.

In this paper we report the establishment of a new carfilzomib-resistant MM cell line derivative, LP-1/Cfz, in which elevated levels of the SQSTM1/p62 ubiquitin-binding cargo receptor were associated with carfilzomib resistance that comprised both prosurvival autophagy and Nrf2 pathway activation. Our results support a model wherein activation of the SQSTM1/p62-Nrf2 pathway — in concert with the PERK-eIF2α/ATF4 axis — directs reprogramming of MM cells, modulating redox and energy homeostasis *via* elevated FAO and inducing prosurvival autophagy involving *GABARAPL1* upregulation [15, 26–29, 34–36]. We suggest that the increased SQSTM1/p62 levels allow newly synthesized Nrf2 to escape Keap1-mediated sequestering in the cytoplasm [13, 14], resulting in the regulation of a subset of its target genes, including *EIF4E3*, which in turn facilitate enhanced translation of Nrf2 and establish a positive feedback loop. While further mechanistic studies are required to fully define the role played by SQSTM1/p62 in carfilzomib resistance, we demonstrated the direct involvement of Nrf2 by genetic and pharmacologic inhibition and a contribution of PERK using two highly selective small-molecule inhibitors of the PERK-eIF2α axis [67, 68].

Previously we reported that acquisition of carfilzomib resistance in KMS-11/Cfz and KMS-34/Cfz MM cells was associated with prosurvival autophagy involving SQSTM1/p62 [11]. Comparative analyses of KMS-11/Cfz cells herein also revealed activation of Nrf2 target genes (Figure 1B). Carfilzomib resistance in KMS-11/Cfz cells is associated with KLF4-mediated transcriptional upregulation of *SQSTM1* expression [11]. Others have described the establishment of a positive feedback loop in which Nrf2 transcriptionally activates *SQSTM1* [13, 14] (highlighted in Figure S1B). We note that *KLF4* is also an Nrf2 target gene (Table S1B), so we envision a model in KMS-11/Cfz cells wherein KLF4 and Nrf2 cooperate to transcriptionally maintain elevated SQSTM1/p62 levels.

GSEA revealed that activation of the PERK-eIF2α axis in LP-1/Cfz cells was not associated with induction of a UPR (Figure S8C). Gupta and colleagues have described a noncanonical mechanism of PERK-Nrf2 activation in the absence of an endoplasmic reticulum stress response that involves an EMT-like adaptation [28, 70]. Enrichment of an EMT-like expression signature was predicted by GSEA for LP-1/Cfz cells (Figure S8D) and for relapsed MM patient samples with increased *EIF4E3* expression (Figure 11C). Further investigation of an EMT-related mechanism is underway and our preliminary studies are suggestive of crosstalk among Nrf2, ERBB2 and hypoxia signaling pathways [82, 83]. Indeed, our ongoing examination of LP-1/Cfz cells indicates that they exhibit intermediate EMT-like characteristics [84]. Of relevance in this regard, a recent study by Orlowski and colleagues identified low levels of *TJP1* encoding the epithelial marker tight junction protein 1 (also known as zonula occludens 1) as a determinant of myeloma proteasome inhibitor resistance [85]. During EMT, intercellular junctions are disrupted and *TJP1* is coordinately downregulated with E-cadherin [86]. Interestingly, *TJP1* expression was decreased in KMS-11/Cfz and KMS-34/Cfz cells but increased in LP-1/Cfz cells (our unpublished results). While Nrf2 has been demonstrated to downregulate E-cadherin expression [72], it has been implicated in the upregulation of *TJP1* expression [87]. Intriguingly, Orlowski and colleagues reported that proteasome inhibitor sensitivity associated with increased *TJP1* expression was due to suppression of expression of the *PSMB8* gene encoding the immunoproteasome β5i/LMP7 (chymotrypsin-like) subunit targeted by carfilzomib and bortezomib as well as the *PSMB9* gene encoding the β1i/LMP2 (caspase-like) subunit that is also targeted by bortezomib [85]. In accord with their findings of an inverse relationship with *TJP1* expression, both *PSMB8* and *PSMB9* were downregulated in LP-1/Cfz cells (Figure S7). In their model, this apparently occurred through TJP1-mediated suppression of EGFR/ERBB2 signaling [85]. As TJP1 also interacts with GABARAPL1 through the CUL3-KBTBD6/KBTBD7 ubiquitin ligase in the autophagy network [53], we speculate that complex interplay between Nrf2-GABARAPL1/TJP1 and ERBB2 signaling pathways may underlie the carfilzomib-resistant LP-1/Cfz phenotype (Figure S3C, S3D) [45, 46, 88, 89].

Carfilzomib resistance in the KMS-11/Cfz and KMS-34/Cfz models was characterized by a partial reversal of plasma cell maturation [11]. These results are in line with the findings of others showing that primary MM cells are capable of dedifferentiating into a less mature MM phenotype conferring experimental and clinical drug resistance [90–92]. It is notable that LP-1/Cfz cells did not fully recapitulate this pattern. Parental LP-1 cells exhibit a membrane phenotype that is intermediate between late B lymphocytes and plasma cells based on MHC class II antigen expression [39]. Like the KMS-11/Cfz and KMS-34/Cfz carfilzomib-resistant MM models [11], acquisition of carfilzomib resistance in LP-1/Cfz cells was accompanied by decreased expression of *SLAMF7* encoding the plasma cell-specific CD319 cell surface marker, reflecting a partial reversal of plasma cell maturation (Table S1A). Conversely, MHC class II antigen expression was diminished, which is reminiscent of plasmacytic differentiation (Table S2A). In this latter respect, LP-1/Cfz cells might resemble in part the situation observed in mantle cell lymphoma where bortezomib resistance increased with plasmacytic differentiation [93]. Accordingly, LP-1/Cfz cells may have attained the “sweet spot” maturation stage intrinsically less susceptible to proteasome inhibition proposed by Tiedemann and colleagues [92].

Our results indicating a role of Nrf2 activation in experimental carfilzomib resistance parallel those of others wherein Nrf2 target gene activation was associated with poor responsiveness to bortezomib in mouse and human MM models [94, 95]. In this regard, both LP-1/Cfz and KMS-11/Cfz exhibit some cross-resistance to bortezomib (Figure S1). In that various regimens were used in the treatment of the MM patients that we analyzed [76, 77, 80], we cannot conclude that increased *EIF4E3* expression can be used as a specific indicator of clinical carfilzomib resistance. Nonetheless, the finding that *EIF4E3* expression is increased in certain chemoresistant minimal residual disease and relapsed MM patient samples and is predictive of Nrf2 target gene activation, strongly suggests that the Nrf2-*EIF4E3* axis and eIF4E3-driven translation contributes to MM drug resistance mechanisms in the clinical setting. On the other hand, lack of *EIF4E3* expression in relapsed samples was able to distinguish a second group of MM patient samples exhibiting the opposite phenotype and pathway activation states in which enhanced eIF4E1-driven translation was indicated. These findings are consistent with the results of other studies demonstrating a role of eIF4E1 in MM biology and proteasome inhibitor resistance [65, 96–98]. It is particularly noteworthy therefore — especially in view of the documented oncogenic activity of *EIF4E1* [75] — that *WHSC1*-expressing MM patients with high *EIF4E3* expression had less favorable outcomes than those with high *EIF4E1* expression (Figure 12).

In summary, our finding of noncanonical SQSTM1/p62-Nrf2 pathway activation adds to the growing appreciation from our work and others that carfilzomib resistance in MM can arise *via* multiple mechanisms [10, 11, 65, 85, 99]. MM is heterogeneous in its etiology and progression so these varied results are not entirely unexpected [100]. Collectively, our studies suggest several approaches to sensitize drug-resistant MM cells to carfilzomib [10, 11]. In particular, the data presented herein support the development of novel therapies targeting the SQSTM1/p62-Nrf2 pathway for a subgroup of advanced stage MM patients with eIF4E3-driven translation.

## MATERIALS AND METHODS

### Cell culture

LP-1 and KMS-11 MM cells were a kind gift from Dr. P. Leif Bergsagel (Mayo Clinic, Scottsdale, AZ) [101]. Cells were cultured in RPMI 1640 with GlutaMAX (Thermo Fisher Scientific) supplemented with 10% fetal bovine serum (Cambrex BioScience), 100 U/ml penicillin and 100 μg/ml streptomycin. Cultures were maintained at 37°C in a humidified atmosphere containing 5% CO_2_.

### Antibodies and reagents

The following antibodies were used: anti-ATF4/CREB-2 (c-20) (Santa Cruz Biotechnology, Cat. No. sc-200); anti-eIF4E1 (P-2) (Santa Cruz Biotechnology, Cat. No. sc-9976); anti-eIF4E3 (Proteintech, Cat. No. 17282-1-AP); anti-FAM129A/Niban (Signalway Antibody, Cat. No. 21401-2); anti-Keap1 (H-190) (Santa Cruz Biotechnology, Cat. No. sc-33569); anti-Nrf2 (H-300) (Santa Cruz Biotechnology, Cat. No. sc-13032); anti-RhoE/Rnd3 clone 4 (EMD Millipore Corporation, Cat. No 05-723); anti-SQSTM1/p62 (Clone 3) mouse mAb (BD Transduction Laboratories, Cat. No. 610832); and anti-α-tubulin mouse mAb (DM1A) (EMD Millipore Corporation, Cat. No. CP06). Carfilzomib (Cat. No. A-1098) was obtained from Active Biochem; chloroquine (Cat. No. S4157) and GSK2656157 (Cat. No. S7033) were purchased from Selleck Chemicals; (S)-4-carboxyphenylglycine (Cat. No. 0323), CGP57380 (Cat. No. 2731), 4EGI-1 (Cat. No. 4800) and MG-132 (Cat. No. 1748) were ordered from Tocris Bioscience; etomoxir (Cat. No. 11969) was obtained from Cayman Chemical; and trigonelline hydrochloride (Cat. No. T5509-1G) was from Sigma-Aldrich.

### Microarray gene expression analysis and quantitative real-time qRT-PCR validation

Total RNA was isolated with the miRNeasy mini kit (Qiagen, Cat. No. 217004). Microarray gene expression analysis of triplicate samples was carried out by Expression Analysis, Inc. (Durham, NC) using Affymetrix GeneChip Human Genome U133 Plus 2.0 arrays. The data have been deposited in GEO (http://www.ncbi.nlm.nih.gov/geo/) under accession number GSE78069. Reverse transcription was performed with the SuperScript VILO cDNA synthesis kit (Thermo Fisher Scientific, Cat. No. 11754250). Real-time qRT-PCR was performed using the Power SYBR Green PCR master mix (Thermo Fisher Scientific, Cat. No. 4367659) on an ABI Prism 7000 Sequence Detection System (Applied Biosystems) as previously described [10, 11]. Primers synthesized by Sigma-Aldrich included: EEF1A2, forward, GTGTACAAGATTGGCGGCAT, reverse, GATGTTCACTGGCGCAAAGG; FAM129A, forward, TACATCCGAGGGAAAACTGAGG, reverse, GCCACAGAGTACTGACGACT; GABARAPL1, forward, AGGAGGACCATCCCTTTGAGT, reverse, TCTACAATCACGGGGACCCT; NFE2L2, forward, CGGTATGCAACAGGACATTG, reverse, TGGCTTCTGGACTTGGAACC; RND3, forward, GTCGGCTGCAAGTCTGATCT, reverse, CCATATTTGCCCCCTGGTCA; and ACTB, forward, GGACTTCGAGCAAGAGATGG, reverse, AGCACTGTGTTGGCGTACAG.

### Chromatin immunoprecipitation (ChIP)

ChIP was performed on the GABARAPL1 promoter region with 20 μg total chromatin and 5 μg of anti-Nrf2 antibody using the SimpleChIP Enzymatic Chromatin IP Kit (Magnetic Beads) (Cell Signaling, Cat. No. 9003) as previously described [11], and 4% of the precipitated material was used per qPCR reaction. Background ChIP levels were obtained using 5 μg of anti-Keap1 antibody. Primers used were: GABARAPL1 promoter region, forward, CCGTGTCCTTCATCTGACTCC, reverse, TCGCTCCTGAACAGCAACAT.

### siRNA transfection

For RNA interference, LP-1/Cfz and parental LP-1 cells were transiently transfected with Nrf2 siRNAs (SI03246950 [#1] and SI03246614 [#2]; QIAGEN, FlexiTube GeneSolution for NFE2L2, Product No. 1027416, Cat. No. GS4780), eIF4E3 siRNAs (s50217 [#1] and s50218 [#2]; Thermo Fisher Scientific Silencer Select) or a negative control siRNA (QIAGEN, ALLStars negative control siRNA, Cat. No. 1027281) using the HiPerFect transfection reagent (QIAGEN). Briefly, 2 × 10^6^ cells per ml were seeded into 24 well plates in 100 μl aliquots. Each well received a mixture of siRNAs (750 ng) and HiPerFect reagent (6 μl) in 100 μl serum-free culture medium preincubated for 15 minutes. After 5 hours, the cells were diluted to 6 × 10^5^ per ml in complete medium. Cells (3 × 10^5^ per ml) were seeded into 96 well plates and treated with a range of carfilzomib concentrations. After 48 hours, cell growth was measured and cells (3 × 10^5^) were lysed for total RNA isolation and qRT-PCR analysis of Nrf2 and GABARAPL1 mRNA levels or western blot analysis of eIF4E3 and Nrf2 protein levels as indicated. The data were normalized to ACTB mRNA levels.

### Cytotoxicity assay

Cells were treated with carfilzomib and agents at the indicated concentrations and cell growth was measured using the alamarBlue cell viability and proliferation reagent (Thermo Fisher Scientific) as previously described [10, 11].

### Measurement of NADPH

NADPH was measured using an NADP/NADPH Quantitation Kit (Sigma-Aldrich, Cat. No. MAK038) according to the manufacturer's instructions [102].

### Measurement of FAO

Oxygen consumption rate was measured using the XF24 Extracellular Flux Analyzer (Seahorse Bioscience) as previously described [103]. FAO was determined by XF Cell Mito Stress Test (Cat. No. 103010-100) using the XF Palmitate-BSA FAO substrate (Cat. No. 102720-100) in the absence or presence of etomoxir according to the manufacturer's instructions.

### ROS and autophagy detection

Cells (4 × 10^5^ per ml) were seeded into 12 well plates in 500 μl aliquots and treated with 0, 25 or 50 nM carfilzomib for 18 hours. H_2_O_2_-associated ROS levels were quantified by flow cytometry using 488 nm excitation and 530/30 nm band pass filter detection after labeling with CM-H_2_DCFDA (Thermo Fisher Scientific, Cat. No. C6827) for 60 minutes at 37°C. O_2_^.−^ levels were quantified by flow cytometry using 488 nm excitation and 585/42 nm band pass detection after labeling with MitoSOX Red (Thermo Fisher Scientific, Cat. No. M36008) for 30 minutes at 37°C. Flow cytometry was performed on a FACSAria instrument equipped with FACSDiva software (BD Biosciences). Dead cells were identified by SYTOX Blue staining (Thermo Fisher Scientific, Cat. No. S34857) and excluded from analysis. Data were analyzed with FlowJo Mac v10.0.2 (Tree Star) and presented on a bivariate plot *versus* 633 nm excitation and 780/60 nm band pass filter detection.

Autophagy was measured with the Cyto-ID autophagy detection kit (Enzo, Cat. No. ENZ-51031-K200) using a FACSAria instrument, and data were analyzed with FlowJo Mac v10.0.2 as previously described [11].

### Cellular fractionation

Nuclear and cytoplasmic extracts were prepared for western blot analysis essentially as described [64].

### Confocal microscopy

Immunofluorescence confocal microscopy was performed as previously described [11]. In brief, cells (2.5 × 10^5^) were centrifuged onto a microscope slide at 1,000 rpm for 5 minutes using a Shandon Cytospin 4 instrument. The cells were then immediately fixed in 3.7% formaldehyde for 5 minutes at room temperature and permeabilized with 0.5% Triton X-100 in phosphate-buffered saline (PBS) for 15 minutes at room temperature. Following permeabilization, the cells were rinsed with PBS and blocked in PBS containing 10% goat serum and 0.01% Triton X-100 for 1 hour at room temperature. The cells were then incubated with anti-Nrf2 antibody diluted to a final concentration of 0.4 μg/ml, in PBS containing 1% goat and 0.01% Triton X-100 for 1 hour at room temperature. The cells were rinsed with PBS and then incubated with Alexa Fluor 488-conjugated goat anti-rabbit secondary antibody (Thermo Fisher Scientific, Cat. No. R37116) diluted 1:500 in PBS containing 1% goat serum and 0.01% Triton X-100 for 1 hour at room temperature. The cells were rinsed with PBS and mounted with Fluoromount G (Electron Microscopy Sciences). Imaging analysis was performed on a Cell Observer SD spinning disk confocal system equipped with Zen software (Carl Zeiss Microscopy).

### Proteasome activity

Proteasome chymotrypsin-like activity was measured by cleavage of a specific luminogenic proteasome substrate (succinyl-leucine-leucine-valine-tyrosine-aminoluciferin) using the Proteasome-Glo Chymotrypsin-Like Assay according to the manufacturer's instructions (Promega Corporation, Cat. No. G8660). Luminescence intensities were quantified with a Gemini XPS microplate spectrofluorometer equipped with SoftMax Pro software (Molecular Devices Corp.).

## SUPPLEMENTARY MATERIAL FIGURES




