# Supplementary material for: Noncanonical SQSTM1/p62-Nrf2 pathway activation mediates proteasome inhibitor resistance in multiple myeloma cells via redox, metabolic and translational reprogramming
Source: Oncotarget. 2016 Sep 10;7(41):66360–85. doi: 10.18632/oncotarget.11960 (PMC5340085; doi:10.18632/oncotarget.11960)
Supplement: Supplementary file 1 [file oncotarget-07-66360-s001.pdf]

Noncanonical SQSTM1/p62-Nrf2 pathway activation mediates proteasome inhibitor resistance in multiple myeloma cells via redox, metabolic and translational reprogramming

Supplementary Material

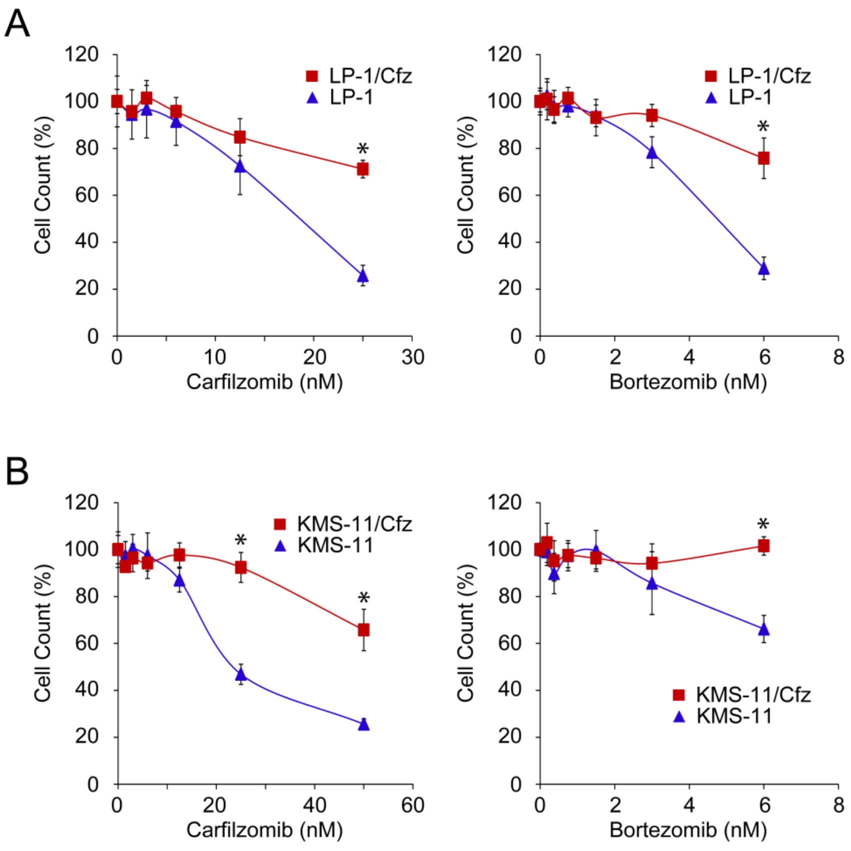

Figure S1: Carfilzomib-resistant LP-1/Cfz and KMS-11/Cfz cells are cross-resistant to bortezomib. A. LP-1/Cfz and parental LP-1 cells were treated with the indicated concentrations of carfilzomib (left graph) or bortezomib (right graph) for 48 hours and cell viability was determined by alamarBlue assay. *, $P < 0.001$ vs parental ($n = 3$). **B.** KMS-11/Cfz and parental KMS-11 cells were treated with the indicated concentrations of carfilzomib (left graph) or bortezomib (right graph) for 48 hours and cell viability was determined by alamarBlue assay. *, $P < 0.001$ vs parental ($n = 3$).

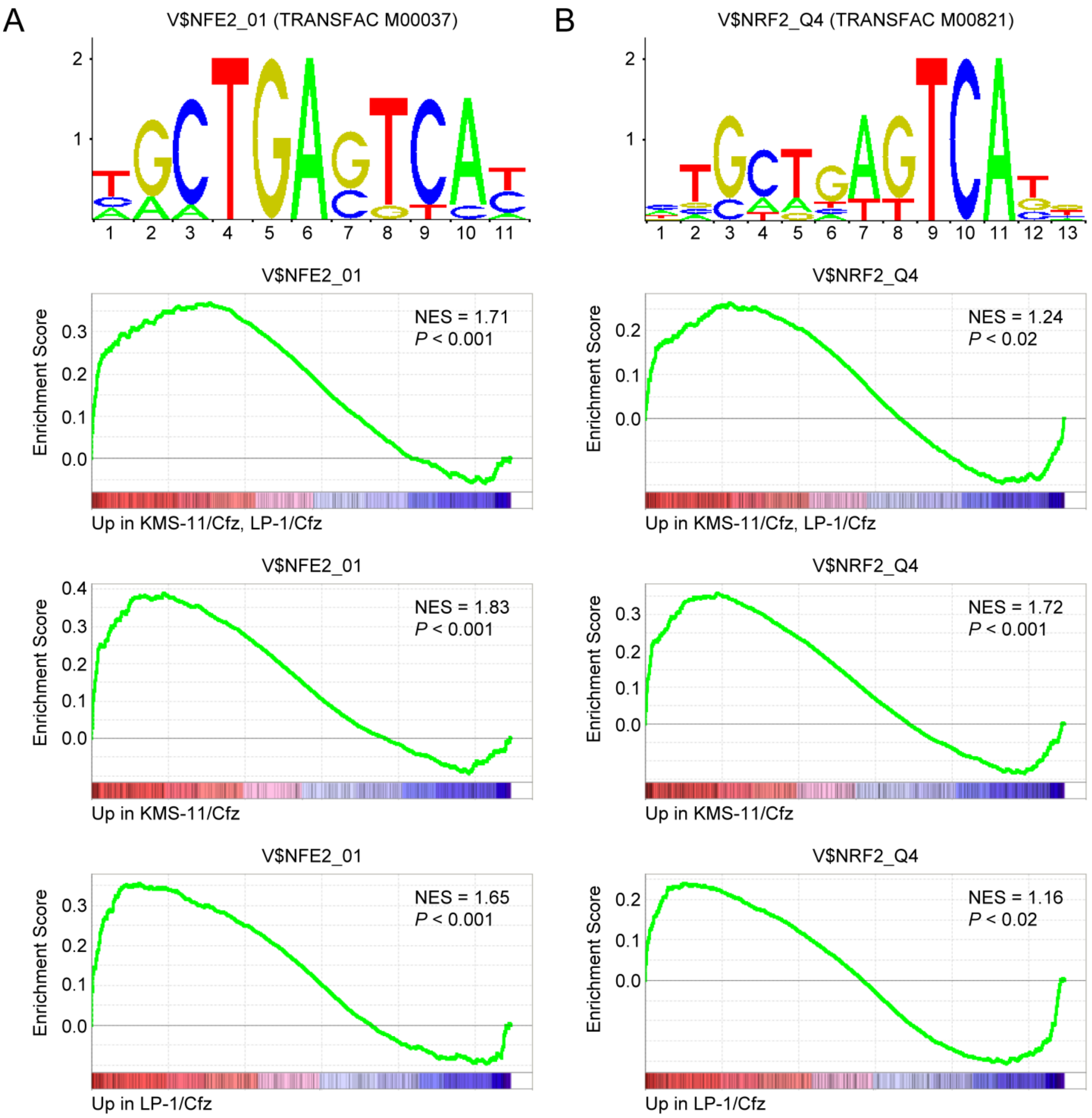
A
V$NFE2_01 (TRANSFAC M00037)
B
V$NRF2_Q4 (TRANSFAC M00821)
V$NFE2_01
Enrichment Score
NES = 1.71
P < 0.001
Up in KMS-11/Cfz, LP-1/Cfz
V$NRF2_Q4
NES = 1.24
P < 0.02
Up in KMS-11/Cfz, LP-1/Cfz
V$NFE2_01
NES = 1.83
P < 0.001
Up in KMS-11/Cfz
V$NRF2_Q4
NES = 1.72
P < 0.001
Up in KMS-11/Cfz
V$NFE2_01
NES = 1.65
P < 0.001
Up in LP-1/Cfz
V$NRF2_Q4
NES = 1.16
P < 0.02
Up in LP-1/Cfz

Figure S2: Coenrichment of NF-E2 and Nrf2 binding site motifs in genes with increased expression in LP-1/Cfz and KMS-11/Cfz cells. A. Sequence logo of the TRANSFAC position weight matrix for NF-E2 (V$NFE2_01; TRANSFAC M00037) and GSEA enrichment plots for pairwise comparisons of carfilzomib-resistant derivatives versus parental cells, combined and individually. Gene set: V$NFE2_01 (M8004). **B.** Sequence logo of the TRANSFAC position weight matrix for Nrf2 (V$NRF2_Q4; TRANSFAC M00821) and GSEA enrichment plots for pairwise comparisons of carfilzomib-resistant derivatives versus parental cells, combined and individually. Gene set: V$NRF2_Q4 (M14141).

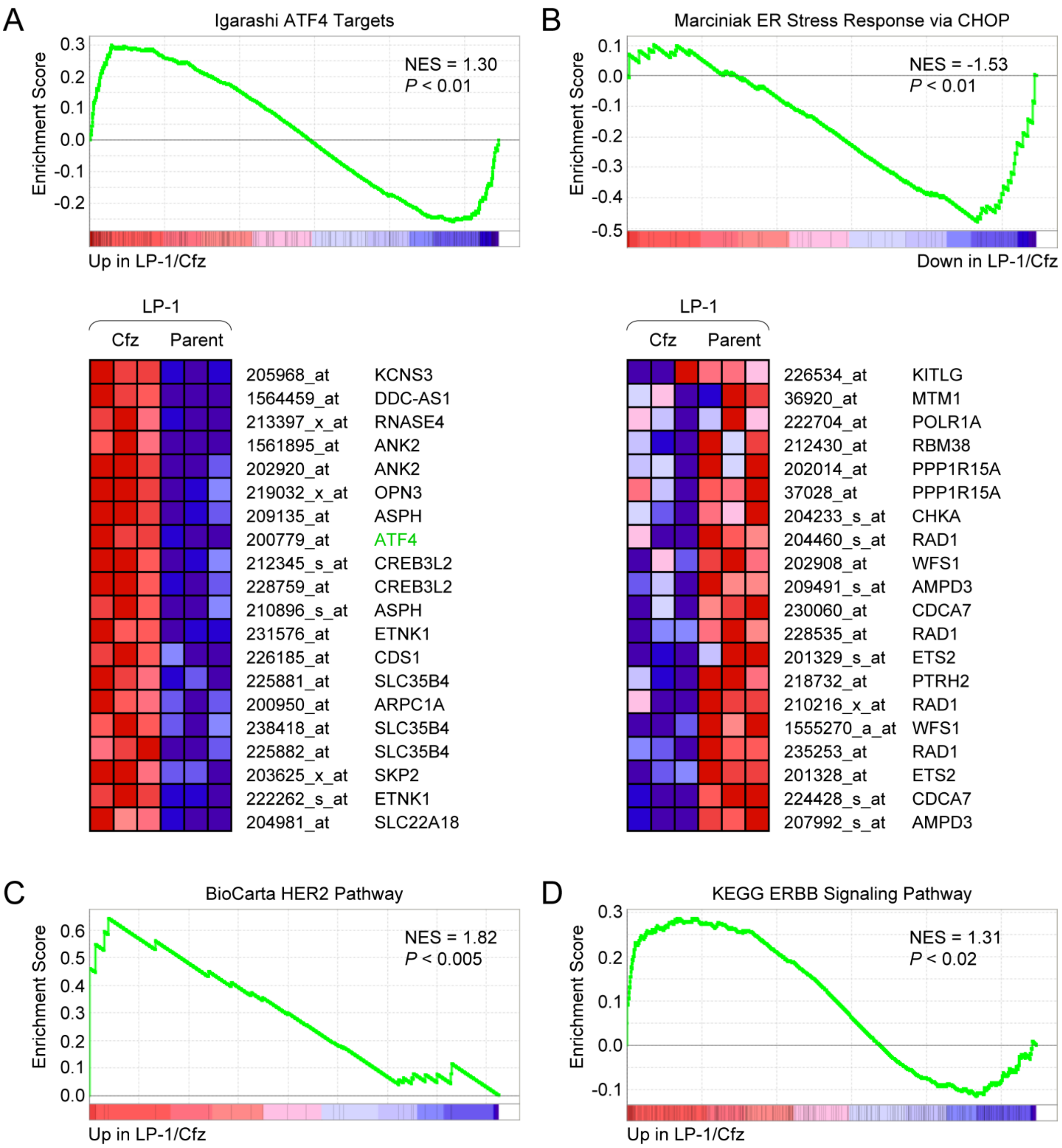
A
Igarashi ATF4 Targets
Enrichment Score
NES = 1.30
P < 0.01
Up in LP-1/Cfz
B
Marciniak ER Stress Response via CHOP
Enrichment Score
NES = -1.53
P < 0.01
Down in LP-1/Cfz
LP-1
Cfz
Parent
205968_at KCNS3
1564459_at DDC-AS1
213397_x_at RNASE4
1561895_at ANK2
202920_at ANK2
219032_x_at OPN3
209135_at ASPH
200779_at ATF4
212345_s_at CREB3L2
228759_at CREB3L2
210896_s_at ASPH
231576_at ETNK1
226185_at CDS1
225881_at SLC35B4
200950_at ARPC1A
238418_at SLC35B4
225882_at SLC35B4
203625_x_at SKP2
222262_s_at ETNK1
204981_at SLC22A18
LP-1
Cfz
Parent
226534_at KITLG
36920_at MTM1
222704_at POLR1A
212430_at RBM38
202014_at PPP1R15A
37028_at PPP1R15A
204233_s_at CHKA
204460_s_at RAD1
202908_at WFS1
209491_s_at AMPD3
230060_at CDCA7
228535_at RAD1
201329_s_at ETS2
218732_at PTRH2
210216_x_at RAD1
1555270_a_at WFS1
235253_at RAD1
201328_at ETS2
224428_s_at CDCA7
207992_s_at AMPD3
C
BioCarta HER2 Pathway
Enrichment Score
NES = 1.82
P < 0.005
Up in LP-1/Cfz
D
KEGG ERBB Signaling Pathway
Enrichment Score
NES = 1.31
P < 0.02
Up in LP-1/Cfz

Figure S3: ATF4 prosurvival and HER2/ERBB2 signatures are enriched in LP-1/Cfz cells. A. GSEA enrichment plot and heat map of the leading edge subset of ATF4 target genes upregulated in LP-1/Cfz (Cfz) versus parental LP-1 (Parent) cells (triplicate samples). A probe set for the *ATF4* gene is highlighted. Gene set: IGARASHI_ATF4_TARGETS_DN, genes down-regulated in A549 cells after knockdown of ATF4 by RNAi (M4779). **B.** GSEA enrichment plot and heat map of the leading edge subset of CHOP target genes downregulated in LP-1/Cfz cells. Gene set: MARCINIAK_ER_STRESS_RESPONSE_VIA_CHOP, endoplasmic reticulum stress response genes dependent on CHOP (M1477). **C., D.** GSEA enrichment plots suggesting upregulation of the HER2/ERBB2 pathway in LP-1/Cfz cells. Gene sets: BIOCARTA_HER2_PATHWAY, role of ERBB2 in signal transduction and oncology (M18719) and KEGG_ERBB_SIGNALING_ PATHWAY (M12467).

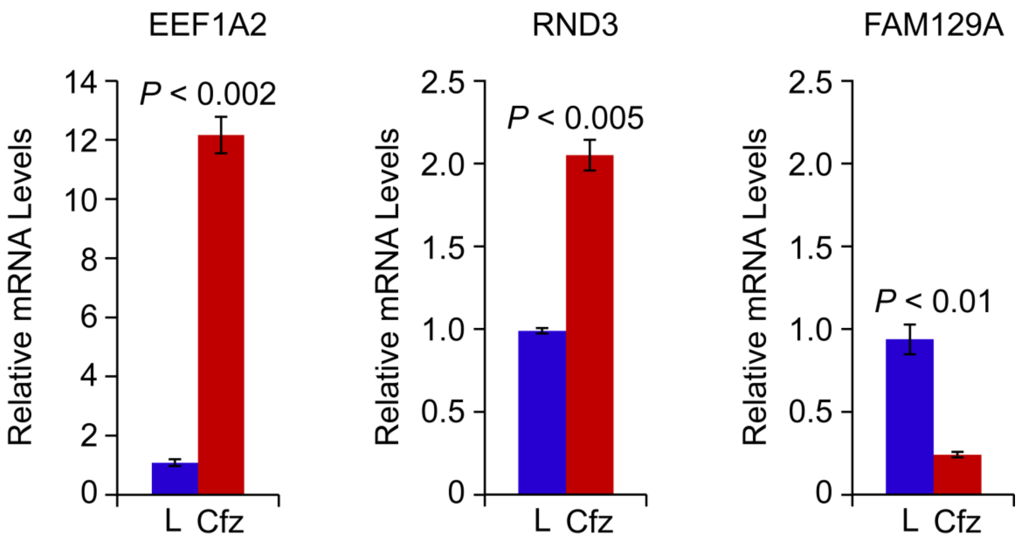

Figure S4: Verification of LP-1/Cfz microarray expression data for selected Nrf2 targets. qRT-PCR analyses were performed to validate the differential expression of *EEF1A2*, *RND3* and *FAM129A* in LP-1/Cfz (Cfz) versus parental LP-1 (L) cells. Mean values of three qRT-PCR experiments. See Tables S1A (*EEF1A2*, *RND3*) and S2A (*FAM129A*) for expression changes determined from the microarray data.

A

Human_GABARAPL1_NM_031412_Promoter_chr12:10364936-10364980_alignment_block_3

```
HUMAN_GABARAPL1_NM_...   tccttcat-ctgact-cctctctt-cagattcctgagtcacgctctg
CHIMP                    tccttcat-ctgact-cctctctt-cagattcctgagtcacgctctg
GORILLA                  tccttcat-ctgact-cctctctt-cagattcctgagtcacgctctg
ORANGUTAN                tccttcat-ctgact-cctctctt-cagattcctgagtcacgctctg
RHESUS                   tccttcat-ctgact-cctctctt-cagattcctgagtcacgctctg
BABOON                   tccttcat-ctgact-cctctctt-cagattcctgagtcacgctctg
MARMOSET                 tccttcac-ctga---catctctt-cagattcctgagtcacgctctg
BUSHBABY                 cccttcac-cttaca-catatttc-aaaattcctgagtcacgctccg
TREESHREW                cccttcac-cttagg-catctctt-caaactcctgagtcacgctctg
GUINEA PIG               cccttcac-------------ctt-caaacgcctgagtcacgctccg
SQUIRREL                 ccctttgc-tttgca-cacccttt-caaattcctgagtcacgctctg
MOUSE                    ttcttcac-cct-ta-cacttctt-caaatccatgagtcatgctctg
RAT                      ttcttcac-cct-ta-cacttctt-caaatccatgagtcacgctgtg
RABBIT                   ------------------------------cccgagtcacgccctg
MOUSE LEMUR              cccttcac-ctgaca-cgtctctc-cgaactcctgagtcacgctgtg
TARSIER                  cccttcac-cttaca-catctctt-caaattcctgagtcacgctctg
DOG                      cccttcag-cttcta-cat---ct-caaattcctgagtcatgctctg
HORSE                    cccttcac-cttata-catctctt-caaattcctgagtcatgctctg
HEDGEHOG                 ccctgcac-tttaaa-catctctt-caaattcctgagtcaagctg--
COW                      cccttcac-cggcta-catctctt-cagttccctgagtcatgctctg
ALPACA                   cccttcag-cttata-cgtctcat-caaatccctgagtcatgctctg
MEGABAT                  cccttcac-ctaata-tgcctttt-ggaattcctgagtcacgttttg
MICROBAT                 cccttcac-ctaata-cgtctctt-cgaattcctgagtcacgatttg
CAT                      cccttcac-cttata-catctcct-caaattcctgagtcatgctctg
ELEPHANT                 cccttcat-attaca-ggtctctt-caaattcctgagtcatgctcag
ROCK HYRAX               cccttcac-cttaca-tatctctt-caaactcctgagtcatgctctg
TENREC                   cccttcacgcttaca-cagctctt-caaattcctgagtcatgctctg
ARMADILLO                cccttcat-cttacg-catctcct-cagatgcctgagtcactctctg
SLOTH                    cccttcat-cgcaggacatcttct-cagattcctgagtcacgctgtg
OPOSSUM                  tctttccc-cgttct-ctccccttccaaaatcctgagtcacgctgtt
PLATYPUS                 ccctcagt-cttcca-cggagcag-aaggtccttgagtcacccgtg
```

position in block: -44 to -1

relative position to TSS (10365488) for reference species: -552 to -509

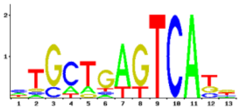

TRANSFAC: NRF2.Q4 (M00821)

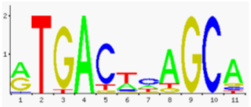

JASPAR: NFE2L2 (MA0150.1)

B

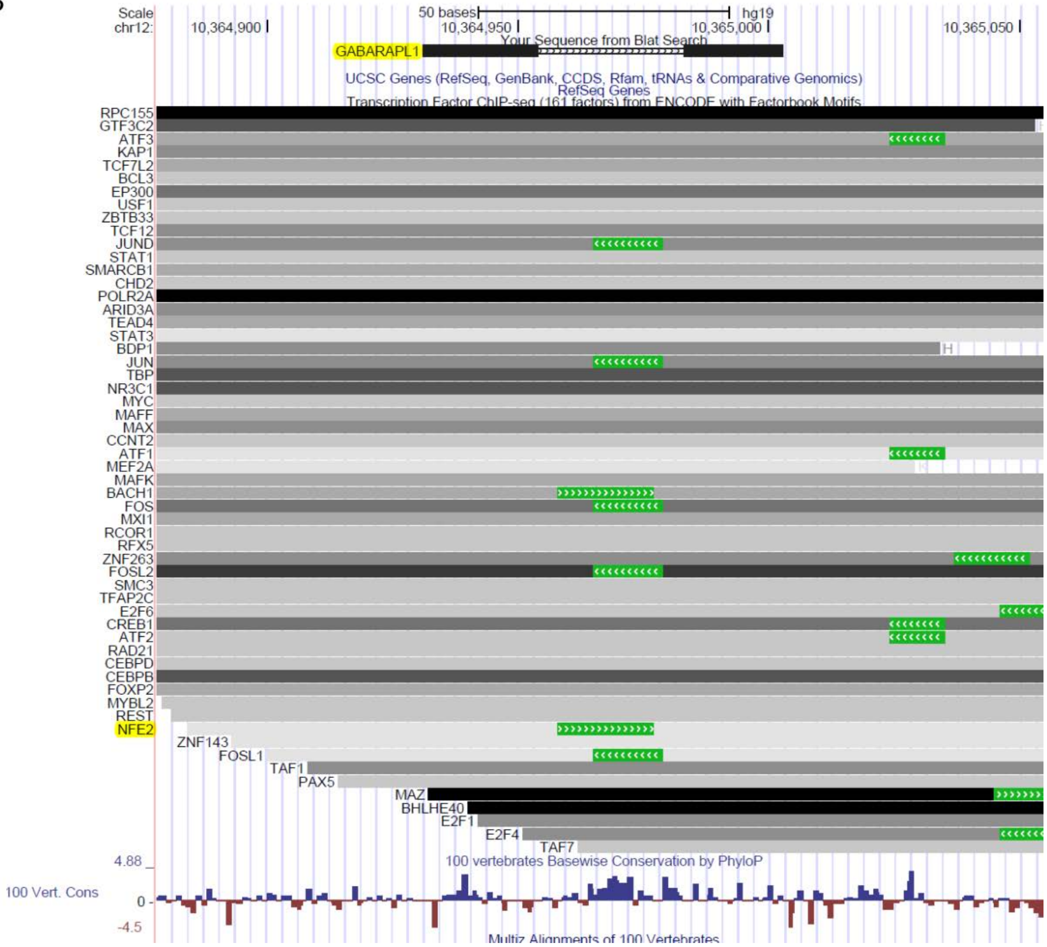

Figure S5: Evolutionarily conserved predicted Nrf2 binding site in the GABARAPL1 promoter region. A. Position of the Nrf2 consensus motifs (TRANSFAC NRF2.Q4 Motif ID, M00821 and JASPAR NFE2L2 Motif ID, MA0150.1) identified in the human GABARAPL1 promoter region (TSS chr12:10365488; NM_031412) using the ConTra v2 transcription factor binding site motif discovery program across species (http://bioit.dmbr.ugent.be/contrav2/ index.php). **B.** Position of Nrf2 ChIP-qPCR primers mapped to the same region of the GABARAPL1 promoter using BLAT in the UCSC genome browser (GRCh37/hg19 assembly). Note that the qPCR primers flank the Nrf2 motif which is an NF-E2 binding site identified by the ENCODE ChIP-seq Project (https://www.encodeproject.org/).

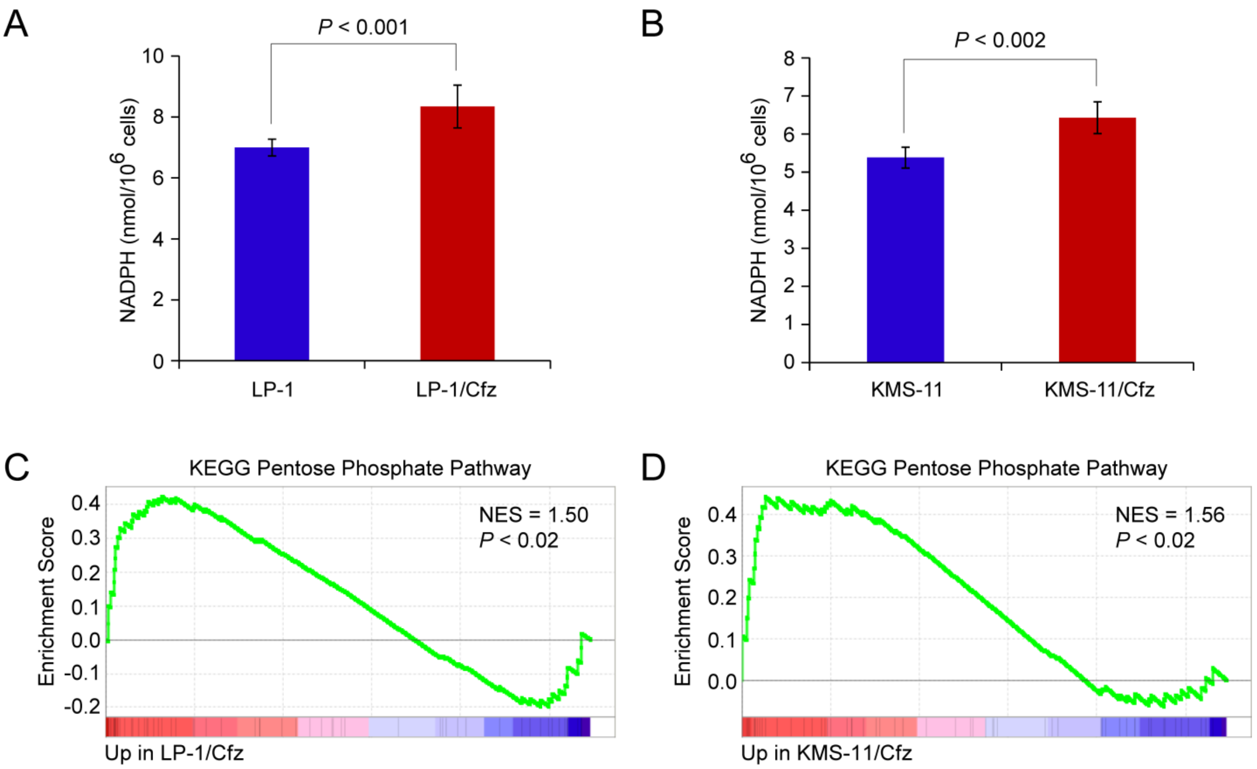

Figure S6: NADPH levels are increased and pentose phosphate pathway genes are upregulated in LP-1/Cfz and KMS-11/Cfz cells. A. NADPH levels in LP-1/Cfz and parental LP-1 cells. **B.** NADPH levels in KMS-11/Cfz and parental KMS-11 cells. **C.** GSEA enrichment plot for triplicate samples of LP-1/Cfz versus parental LP-1 cells. Gene set: KEGG pentose phosphate pathway (M1386; KEGG Pathway hsa00030). **D.** GSEA enrichment plot for triplicate samples of KMS-11/Cfz versus parental KMS-11 cells. Gene set: KEGG pentose phosphate pathway (M1386; KEGG Pathway hsa00030).

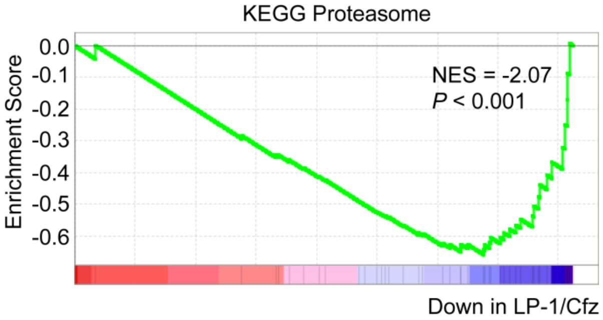

KEGG Proteasome
Enrichment Score
0.0
-0.1
-0.2
-0.3
-0.4
-0.5
-0.6
NES = -2.07
P < 0.001
Down in LP-1/Cfz

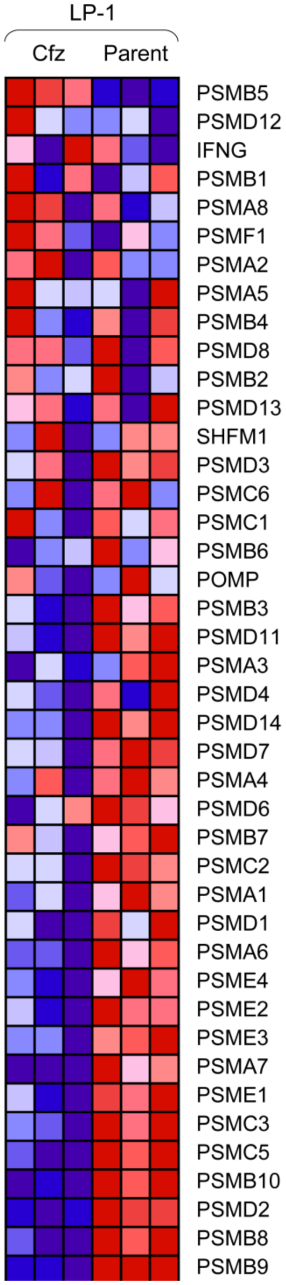

LP-1
Cfz
Parent
PSMB5
PSMD12
IFNG
PSMB1
PSMA8
PSMF1
PSMA2
PSMA5
PSMB4
PSMD8
PSMB2
PSMD13
SHFM1
PSMD3
PSMC6
PSMC1
PSMB6
POMP
PSMB3
PSMD11
PSMA3
PSMD4
PSMD14
PSMD7
PSMA4
PSMD6
PSMB7
PSMC2
PSMA1
PSMD1
PSMA6
PSME4
PSME2
PSME3
PSMA7
PSME1
PSMC3
PSMC5
PSMB10
PSMD2
PSMB8
PSMB9

Figure S7: GSEA enrichment plot and heat map showing downregulation of 19S proteasome subunit genes in LP-1/Cfz cells. GSEA enrichment plot and heat map of gene expression changes for triplicate samples of LP-1/Cfz (Cfz) and LP-1 (Parent) cells. Gene set: KEGG proteasome (KEGG Pathway hsa03050; M10680). Whereas mRNA levels of *PSMB5* encoding the β5 catalytic subunit of the 20S proteasome targeted by carfilzomib were modestly increased in LP-1/Cfz cells, mRNA levels of *PSMB8* encoding the immunoproteasome β5i/LMP7 subunit that is also specifically targeted by carfilzomib and those of many of the 19S (*PSMC2*, *PSMC3*, *PSMC5*, *PSMD1*, *PSMD14*, *PSMD2*, *PSMD6* and *PSMD7*) and 11S (*PSME1*, *PSME2*, *PSME3* and *PSME4*) proteasome subunit genes were decreased.

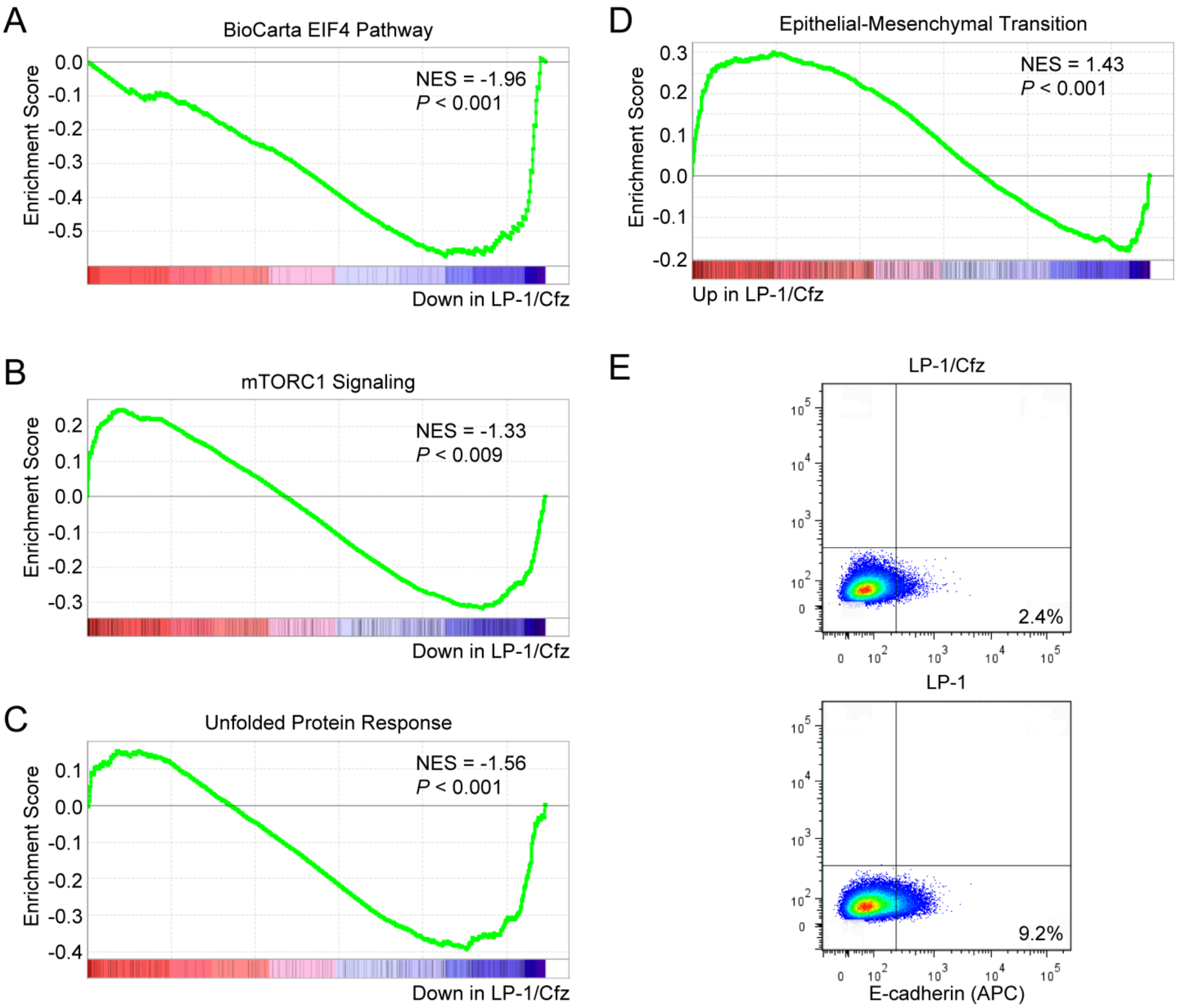

A
BioCarta EIF4 Pathway
Enrichment Score
0.0
-0.1
-0.2
-0.3
-0.4
-0.5
NES = -1.96
P < 0.001
Down in LP-1/Cfz
B
mTORC1 Signaling
Enrichment Score
0.2
0.1
0.0
-0.1
-0.2
-0.3
NES = -1.33
P < 0.009
Down in LP-1/Cfz
C
Unfolded Protein Response
Enrichment Score
0.1
0.0
-0.1
-0.2
-0.3
-0.4
NES = -1.56
P < 0.001
Down in LP-1/Cfz
D
Epithelial-Mesenchymal Transition
Enrichment Score
0.3
0.2
0.1
0.0
-0.1
-0.2
NES = 1.43
P < 0.001
Up in LP-1/Cfz
E
LP-1/Cfz
2.4%
LP-1
9.2%
E-cadherin (APC)

Figure S8: GSEA suggests altered translational regulation in LP-1/Cfz cells and acquisition of EMT-like features. A. GSEA enrichment plot showing downregulation of EIF4 pathway genes in LP-1/Cfz cells. Gene set: BIOCARTA_EIF4_PATHWAY, regulation of eIF4E and p70 S6 kinase (M4791). **B.** GSEA enrichment plot showing downregulation of mTORC1 signaling in LP-1/Cfz cells. Gene set: HALLMARK_MTORC1_SIGNALING. **C.** GSEA indicated that genes upregulated during the UPR are downregulated in LP-1/Cfz cells Gene set: HALLMARK_UNFOLDED_PROTEIN_ RESPONSE (M5922). **D.** GSEA indicated enrichment of an EMT-like expression signature in LP-1/Cfz cells. Gene set: HALLMARK_EPITHELIAL_MESENCHYMAL_TRANSITION (M5930). **E.** Decreased cell surface expression of E-cadherin on LP-1/Cfz cells compared to parental LP-1 cells determined by flow cytometry after staining with an allophycocyanin (APC)-conjugated anti-E-cadherin antibody. Percentages of E-cadherin-positive cells are indicated.

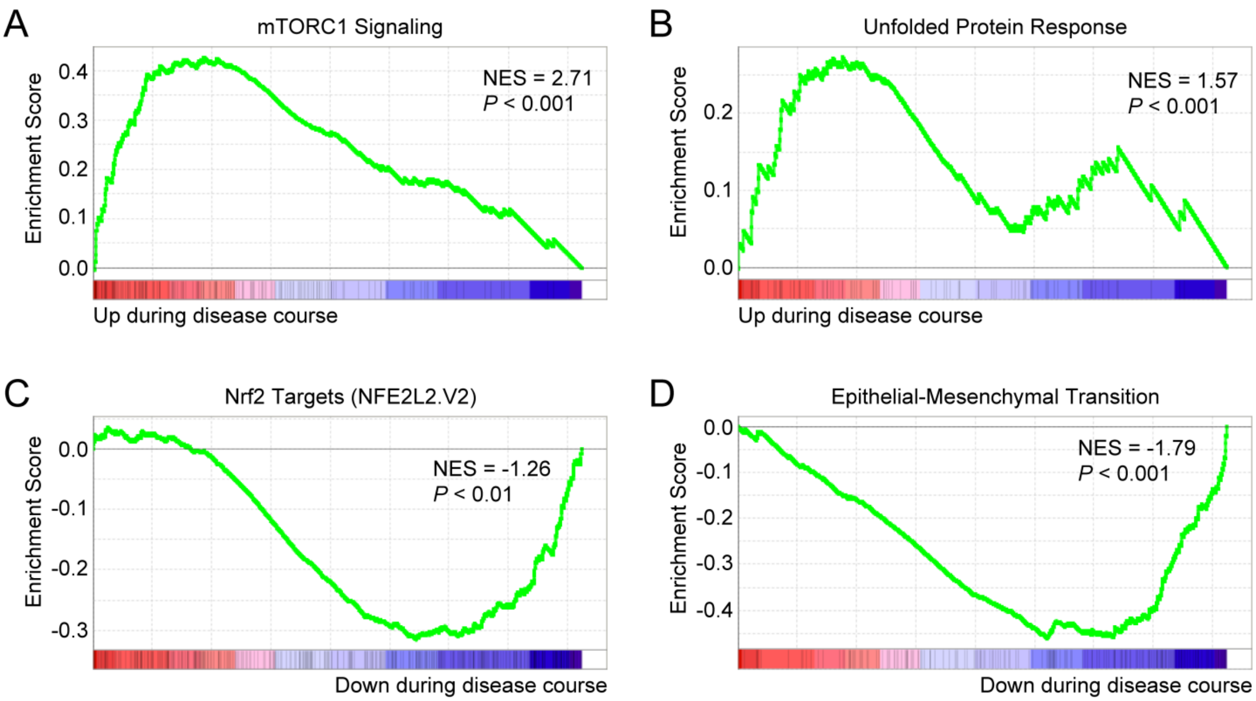

Figure S9: Gene expression signatures of MM cells without increased *EIF4E3* and/or *GABARAPL1* expression during progression of disease. Microarray data was obtained from the GEO database (GEO accession number GSE36824) for patient-paired relapse and diagnostic samples from 17 patients treated with various regimens. GSEA was performed on the samples from 10 patients where *EIF4E3* and/or *GABARAPL1* expression was not increased during disease course. **A.** Genes upregulated through activation of the mTORC1 complex were upregulated in these cases. Gene set: HALLMARK_MTORC1_ SIGNALING (M5924). **B.** GSEA indicated enrichment for genes upregulated during the UPR. Gene set: HALLMARK_UNFOLDED_PROTEIN_ RESPONSE (M5922). **C.** GSEA indicated that Nrf2 target genes were downregulated during disease course in these cases. Gene set: NFE2L2.V2 (M2870). **D.** These patients had decreased enrichment of an EMT-like expression signature Gene set: HALLMARK_EPITHELIAL_ MESENCHYMAL_TRANSITION (M5930).
