# Supplementary material for: Noncanonical SQSTM1/p62-Nrf2 pathway activation mediates proteasome inhibitor resistance in multiple myeloma cells via redox, metabolic and translational reprogramming
Source: Oncotarget. 2016 Sep 10;7(41):66360–85. doi: 10.18632/oncotarget.11960 (PMC5340085; doi:10.18632/oncotarget.11960)
Supplement: Supplementary file 2 [file oncotarget-07-66360-s002.docx]

**Supplementary Tables**

**Table S1A. Differentially expressed Nrf2 target genes in LP-1/Cfz versus LP-1 cells**

| **Gene Symbol** | **Gene Name** | **Probe Set ID** | **Fold Change** |
| --- | --- | --- | --- |
| EEF1A2 | eukaryotic translation elongation factor 1 alpha 2 | 204540_at | 3.63 |
| ADSSL1 | adenylosuccinate synthase like 1 | 226325_at | 3.60 |
| GPC5 | glypican 5 | 207174_at | 3.53 |
| CAP2 | CAP, adenylate cyclase-associated protein, 2 (yeast) | 212554_at | 2.93 |
| RND3 | Rho family GTPase 3 | 212724_at | 2.62 |
| CAP2 | CAP, adenylate cyclase-associated protein, 2 (yeast) | 212551_at | 2.52 |
| TANC2 | tetratricopeptide repeat, ankyrin repeat and coiled-coil containing 2 | 224952_at | 2.36 |
| GBE1 | glucan (1,4-alpha-), branching enzyme 1 | 203282_at | 2.14 |
| GABARAPL1 | GABA(A) receptor-associated protein like 1 | 208869_s_at | 2.14 |
| GABARAPL1/GABARAPL3 | GABA(A) receptor-associated protein like 1/GABA(A) receptors associated protein like 3, pseudogene | 211458_s_at | 2.12 |
| SLC7A11 | solute carrier family 7 (anionic amino acid transporter light chain, xc- system), member 11 | 217678_at | 2.02 |
| PTPRM | protein tyrosine phosphatase, receptor type, M | 1555579_s_at | 1.94 |
| EIF4E3 | eukaryotic translation initiation factor 4E family member 3 | 225941_at | 1.93 |
| SLC7A11 | solute carrier family 7 (anionic amino acid transporter light chain, xc- system), member 11 | 209921_at | 1.92 |
| DST | dystonin | 204455_at | 1.88 |
| DST | dystonin | 232098_at | 1.78 |
| PAM | peptidylglycine alpha-amidating monooxygenase | 202336_s_at | 1.77 |
| GPC6 | glypican 6 | 227059_at | 1.76 |
| CRIM1 | cysteine rich transmembrane BMP regulator 1 (chordin-like) | 202551_s_at | 1.76 |
| FOXD1 | forkhead box D1 | 206307_s_at | 1.75 |
| EIF4E3 | eukaryotic translation initiation factor 4E family member 3 | 238461_at | 1.74 |
| YES1 | v-yes-1 Yamaguchi sarcoma viral oncogene homolog 1 | 202933_s_at | 1.74 |
| PAM | peptidylglycine alpha-amidating monooxygenase | 214620_x_at | 1.73 |
| INF2 | inverted formin, FH2 and WH2 domain containing | 222534_s_at | 1.71 |
| EYA1 | eyes absent homolog 1 (Drosophila) | 214608_s_at | 1.70 |
| CR2 | complement component (3d/Epstein Barr virus) receptor 2 | 205544_s_at | 1.69 |
| BTG1 | B-cell translocation gene 1, anti-proliferative | 200920_s_at | -1.69 |
| BTG1 | B-cell translocation gene 1, anti-proliferative | 200921_s_at | -1.69 |
| PHLDA1 | pleckstrin homology-like domain, family A, member 1 | 217996_at | -1.69 |
| PIK3CG | phosphatidylinositol-4,5-bisphosphate 3-kinase, catalytic subunit gamma | 206369_s_at | -1.69 |
| IVD | isovaleryl-CoA dehydrogenase | 203682_s_at | -1.70 |
| JAG1 | jagged 1 | 216268_s_at | -1.72 |
| CHST11 | carbohydrate (chondroitin 4) sulfotransferase 11 | 226368_at | -1.73 |
| ADD3 | adducin 3 (gamma) | 201034_at | -1.74 |
| IVD | isovaleryl-CoA dehydrogenase | 216958_s_at | -1.75 |
| APOL6 | apolipoprotein L, 6 | 219716_at | -1.77 |
| ANK3 | ankyrin 3, node of Ranvier (ankyrin G) | 206385_s_at | -1.78 |
| PHLDA1 | pleckstrin homology-like domain, family A, member 1 | 217997_at | -1.80 |
| ST8SIA4 | ST8 alpha-N-acetyl-neuraminide alpha-2,8-sialyltransferase 4 | 206925_at | -1.80 |
| IVD | isovaleryl-CoA dehydrogenase | 225311_at | -1.81 |
| JAG1 | jagged 1 | 209099_x_at | -1.83 |
| CDKN2B | cyclin-dependent kinase inhibitor 2B (p15, inhibits CDK4) | 236313_at | -1.83 |
| CBLB | Cbl proto-oncogene, E3 ubiquitin protein ligase B | 209682_at | -1.83 |
| PCDH9 | protocadherin 9 | 219737_s_at | -1.85 |
| ADD3 | adducin 3 (gamma) | 201753_s_at | -1.86 |
| PTPRK | protein tyrosine phosphatase, receptor type, K | 203038_at | -1.87 |
| ETS1 | v-ets erythroblastosis virus E26 oncogene homolog 1 (avian) | 224833_at | -1.88 |
| ENC1 | ectodermal-neural cortex 1 (with BTB domain) | 201340_s_at | -1.88 |
| CCDC104 | coiled-coil domain containing 104 | 224968_at | -1.89 |
| DUSP4 | dual specificity phosphatase 4 | 204014_at | -1.93 |
| CTSO | cathepsin O | 203758_at | -1.93 |
| CHST11 | carbohydrate (chondroitin 4) sulfotransferase 11 | 219634_at | -1.95 |
| UTRN | utrophin | 225093_at | -1.96 |
| PDGFC | platelet derived growth factor C | 222719_s_at | -1.96 |
| SGK1 | serum/glucocorticoid regulated kinase 1 | 201739_at | -1.97 |
| FERMT3 | fermitin family member 3 | 223303_at | -1.97 |
| ADD3 | adducin 3 (gamma) | 201752_s_at | -2.00 |
| NETO2 | neuropilin (NRP) and tolloid (TLL)-like 2 | 222774_s_at | -2.00 |
| ADD3 | adducin 3 (gamma) | 205882_x_at | -2.03 |
| CEP70 | centrosomal protein 70kDa | 1554489_a_at | -2.09 |
| CEP70 | centrosomal protein 70kDa | 219036_at | -2.14 |
| CDC42EP3 | CDC42 effector protein (Rho GTPase binding) 3 | 209286_at | -2.14 |
| ETS1 | v-ets erythroblastosis virus E26 oncogene homolog 1 (avian) | 1555355_a_at | -2.14 |
| DUSP5 | dual specificity phosphatase 5 | 209457_at | -2.17 |
| CEP70 | centrosomal protein 70kDa | 224150_s_at | -2.24 |
| APOL6 | apolipoprotein L, 6 | 241869_at | -2.24 |
| PLEK | pleckstrin | 203471_s_at | -2.24 |
| CPT1A | carnitine palmitoyltransferase 1A (liver) | 203634_s_at | -2.26 |
| FZD6 | frizzled family receptor 6 | 203987_at | -2.27 |
| FTL | ferritin, light polypeptide | 212788_x_at | -2.28 |
| PELI2 | pellino E3 ubiquitin protein ligase family member 2 | 219132_at | -2.30 |
| CEP70 | centrosomal protein 70kDa | 238154_at | -2.32 |
| FNBP1 | formin binding protein 1 | 212288_at | -2.33 |
| ST8SIA4 | ST8 alpha-N-acetyl-neuraminide alpha-2,8-sialyltransferase 4 | 242943_at | -2.38 |
| RASGRP3 | RAS guanyl releasing protein 3 (calcium and DAG-regulated) | 205801_s_at | -2.39 |
| ST8SIA4 | ST8 alpha-N-acetyl-neuraminide alpha-2,8-sialyltransferase 4 | 230261_at | -2.47 |
| IFIT3 | interferon-induced protein with tetratricopeptide repeats 3 | 229450_at | -2.52 |
| CHST11 | carbohydrate (chondroitin 4) sulfotransferase 11 | 226372_at | -2.65 |
| RTP4 | receptor (chemosensory) transporter protein 4 | 219684_at | -2.66 |
| FCHSD2 | FCH and double SH3 domains 2 | 203620_s_at | -2.66 |
| ARHGDIB | Rho GDP dissociation inhibitor (GDI) beta | 201288_at | -2.67 |
| BLK | B lymphoid tyrosine kinase | 206255_at | -2.72 |
| MSANTD3-TMEFF1/ TMEFF1 | MSANTD3-TMEFF1 readthrough/transmembrane protein with EGF-like and two follistatin-like domains 1 | 205122_at | -2.76 |
| ARHGDIB | Rho GDP dissociation inhibitor (GDI) beta | 1555812_a_at | -2.90 |
| FTL | ferritin, light polypeptide | 213187_x_at | -2.90 |
| RAI14 | retinoic acid induced 14 | 202052_s_at | -2.93 |
| CPT1A | carnitine palmitoyltransferase 1A (liver) | 203633_at | -3.31 |
| CDC42EP3 | CDC42 effector protein (Rho GTPase binding) 3 | 225685_at | -3.34 |
| TWIST1 | twist basic helix-loop-helix transcription factor 1 | 213943_at | -3.49 |
| TGIF1 | TGFB-induced factor homeobox 1 | 203313_s_at | -3.68 |
| ST8SIA4 | ST8 alpha-N-acetyl-neuraminide alpha-2,8-sialyltransferase 4 | 230836_at | -3.72 |
| MSANTD3-TMEFF1/ TMEFF1 | MSANTD3-TMEFF1 readthrough/transmembrane protein with EGF-like and two follistatin-like domains 1 | 205123_s_at | -3.73 |
| ZFPM2 | zinc finger protein, FOG family member 2 | 219778_at | -3.93 |
| SLAMF7 | SLAM family member 7 | 219159_s_at | -4.23 |
| P2RY10 | purinergic receptor P2Y, G-protein coupled, 10 | 1553856_s_at | -4.25 |
| SLAMF7 | SLAM family member 7 | 234306_s_at | -4.42 |
| CDC42EP3 | CDC42 effector protein (Rho GTPase binding) 3 | 209288_s_at | -4.44 |
| CCDC109B | coiled-coil domain containing 109B | 218802_at | -4.63 |
| SLAMF7 | SLAM family member 7 | 222838_at | -4.87 |
| IGFBP7 | insulin-like growth factor binding protein 7 | 201162_at | -8.65 |
| IGFBP7 | insulin-like growth factor binding protein 7 | 201163_s_at | -18.27 |
| PRDX1 | peroxiredoxin 1 | 208680_at | -66.20 |

**Table S1B. Differentially expressed Nrf2 target genes in KMS-11/Cfz versus KMS-11 cells**

| **Gene Symbol** | **Gene Name** | **Probe Set ID** | **Fold Change** |
| --- | --- | --- | --- |
| BLVRB | biliverdin reductase B (flavin reductase (NADPH)) | 202201_at | 3.66 |
| CYP39A1 | cytochrome P450, family 39, subfamily A, polypeptide 1 | 244407_at | 2.88 |
| PIR | pirin (iron-binding nuclear protein) | 207469_s_at | 2.62 |
| GABARAPL1/GABARAPL3 | GABA(A) receptor-associated protein like 1/GABA(A) receptors associated protein like 3, pseudogene | 211458_s_at | 2.34 |
| GABARAPL1 | GABA(A) receptor-associated protein like 1 | 208869_s_at | 2.30 |
| CYP39A1 | cytochrome P450, family 39, subfamily A, polypeptide 1 | 220432_s_at | 2.04 |
| PPAP2C | phosphatidic acid phosphatase type 2C | 209529_at | 1.96 |
| GABARAPL1 | GABA(A) receptor-associated protein like 1 | 208868_s_at | 1.86 |
| LILRB4 | leukocyte immunoglobulin-like receptor, subfamily B (with TM and ITIM domains), member 4 | 210152_at | 1.85 |
| GPNMB | glycoprotein (transmembrane) nmb | 201141_at | 1.82 |
| DENND5A | DENN/MADD domain containing 5A | 212561_at | 1.72 |
| RASSF6 | Ras association (RalGDS/AF-6) domain family member 6 | 229147_at | 1.70 |
| KLF4 | Kruppel-like factor 4 (gut) | 221841_s_at | 1.66 |
| SQSTM1 | sequestosome 1 | 213112_s_at | 1.64 |
| SQSTM1 | sequestosome 1 | 201471_s_at | 1.62 |
| CYP39A1 | cytochrome P450, family 39, subfamily A, polypeptide 1 | 1553977_a_at | 1.60 |
| TALDO1 | transaldolase 1 | 201463_s_at | 1.58 |
| GCLM | glutamate-cysteine ligase, modifier subunit | 234986_at | 1.58 |
| IFIT3 | interferon-induced protein with tetratricopeptide repeats 3 | 229450_at | 1.56 |
| AIFM2 | apoptosis-inducing factor, mitochondrion-associated, 2 | 228445_at | 1.54 |
| ICK | intestinal cell (MAK-like) kinase | 204569_at | 1.53 |
| ADCY7 | adenylate cyclase 7 | 203741_s_at | 1.52 |
| NQO2 | NAD(P)H dehydrogenase, quinone 2 | 203814_s_at | 1.52 |
| IFIT3 | interferon-induced protein with tetratricopeptide repeats 3 | 204747_at | 1.51 |
| GSTA4 | glutathione S-transferase alpha 4 | 235405_at | 1.51 |
| LMNA | lamin A/C | 1554600_s_at | 1.50 |
| AIFM2 | apoptosis-inducing factor, mitochondrion-associated, 2 | 224461_s_at | 1.49 |
| OSGIN1 | oxidative stress induced growth inhibitor 1 | 219475_at | 1.49 |
| CREM | cAMP responsive element modulator | 209967_s_at | 1.49 |
| LMNA | lamin A/C | 203411_s_at | 1.49 |
| ABCB6 | ATP-binding cassette, sub-family B (MDR/TAP), member 6 | 203192_at | 1.49 |
| NIT2 | nitrilase family, member 2 | 218557_at | 1.48 |
| SQSTM1 | sequestosome 1 | 244804_at | 1.47 |
| LRP12 | low density lipoprotein receptor-related protein 12 | 220253_s_at | 1.46 |
| NQO1 | NAD(P)H dehydrogenase, quinone 1 | 201467_s_at | 1.45 |
| CREM | cAMP responsive element modulator | 228092_at | 1.45 |
| DPYD | dihydropyrimidine dehydrogenase | 1554534_at | 1.45 |
| PIK3CD | phosphatidylinositol-4,5-bisphosphate 3-kinase, catalytic subunit delta | 203879_at | 1.44 |
| FAM43A | family with sequence similarity 43, member A | 227410_at | 1.43 |
| TCF4 | transcription factor 4 | 228837_at | 1.43 |
| AUTS2 | autism susceptibility candidate 2 | 212599_at | 1.43 |
| CBR3 | carbonyl reductase 3 | 205379_at | 1.43 |
| PTPRM | protein tyrosine phosphatase, receptor type, M | 203329_at | 1.43 |
| CTSB | cathepsin B | 200838_at | 1.42 |
| GCLM | glutamate-cysteine ligase, modifier subunit | 236140_at | 1.41 |
| LRP12 | low density lipoprotein receptor-related protein 12 | 219631_at | 1.41 |
| CBLB | Cbl proto-oncogene, E3 ubiquitin protein ligase B | 209682_at | 1.40 |
| SRXN1 | sulfiredoxin 1 | 225252_at | 1.40 |
| CTSB | cathepsin B | 227961_at | 1.40 |
| SCPEP1 | serine carboxypeptidase 1 | 218217_at | 1.40 |
| NQO1 | NAD(P)H dehydrogenase, quinone 1 | 210519_s_at | 1.40 |
| LRP12 | low density lipoprotein receptor-related protein 12 | 220254_at | 1.39 |
| CTSB | cathepsin B | 213274_s_at | 1.39 |
| ZYX | zyxin | 200808_s_at | 1.38 |
| TGIF1 | TGFB-induced factor homeobox 1 | 1566901_at | 1.38 |
| CREM | cAMP responsive element modulator | 230511_at | 1.38 |
| HHEX | hematopoietically expressed homeobox | 204689_at | 1.37 |
| HTATIP2 | HIV-1 Tat interactive protein 2, 30kDa | 209448_at | 1.37 |
| RABL2A/RABL2B | RAB, member of RAS oncogene family-like 2A/RAB, member of RAS oncogene family-like 2B | 219151_s_at | 1.36 |
| POU4F1 | POU class 4 homeobox 1 | 211341_at | -1.36 |
| PDIA4 | protein disulfide isomerase family A, member 4 | 208658_at | -1.36 |
| PHLDA1 | pleckstrin homology-like domain, family A, member 1 | 217999_s_at | -1.36 |
| PHLDA1 | pleckstrin homology-like domain, family A, member 1 | 217997_at | -1.36 |
| IDH1 | isocitrate dehydrogenase 1 (NADP+), soluble | 1555037_a_at | -1.37 |
| TLE3 | transducin-like enhancer of split 3 (E(sp1) homolog, Drosophila) | 212769_at | -1.37 |
| ARHGAP18 | Rho GTPase activating protein 18 | 225173_at | -1.39 |
| CADM1 | cell adhesion molecule 1 | 209031_at | -1.39 |
| SLC12A8 | solute carrier family 12 (potassium/chloride transporters), member 8 | 219874_at | -1.40 |
| FCHSD2 | FCH and double SH3 domains 2 | 203620_s_at | -1.42 |
| PRKCB | protein kinase C, beta | 209685_s_at | -1.42 |
| BFSP2 | beaded filament structural protein 2, phakinin | 207399_at | -1.42 |
| LRRK1 | leucine-rich repeat kinase 1 | 219441_s_at | -1.42 |
| IGF1 | insulin-like growth factor 1 (somatomedin C) | 211577_s_at | -1.42 |
| SIPA1L2 | signal-induced proliferation-associated 1 like 2 | 233587_s_at | -1.43 |
| SEC61B | Sec61 beta subunit | 244700_at | -1.44 |
| SSR3 | signal sequence receptor, gamma (translocon-associated protein gamma) | 217790_s_at | -1.44 |
| CADM1 | cell adhesion molecule 1 | 209030_s_at | -1.44 |
| ABHD2 | abhydrolase domain containing 2 | 228490_at | -1.45 |
| IGF1 | insulin-like growth factor 1 (somatomedin C) | 209542_x_at | -1.45 |
| CADM1 | cell adhesion molecule 1 | 209032_s_at | -1.47 |
| ITGA8 | integrin, alpha 8 | 214265_at | -1.48 |
| SLC1A4 | solute carrier family 1 (glutamate/neutral amino acid transporter), member 4 | 209611_s_at | -1.48 |
| GBE1 | glucan (1,4-alpha-), branching enzyme 1 | 203282_at | -1.48 |
| ABHD2 | abhydrolase domain containing 2 | 205566_at | -1.55 |
| IGF1 | insulin-like growth factor 1 (somatomedin C) | 209541_at | -1.58 |
| NAV1 | neuron navigator 1 | 224774_s_at | -1.58 |
| SLC1A4 | solute carrier family 1 (glutamate/neutral amino acid transporter), member 4 | 212810_s_at | -1.59 |
| ITGA8 | integrin, alpha 8 | 235666_at | -1.61 |
| NAV1 | neuron navigator 1 | 224773_at | -1.61 |
| P4HA1 | prolyl 4-hydroxylase, alpha polypeptide I | 207543_s_at | -1.63 |
| IGF1 | insulin-like growth factor 1 (somatomedin C) | 209540_at | -1.65 |
| NAV1 | neuron navigator 1 | 224772_at | -1.65 |
| SLC1A4 | solute carrier family 1 (glutamate/neutral amino acid transporter), member 4 | 212811_x_at | -1.69 |
| SLC1A4 | solute carrier family 1 (glutamate/neutral amino acid transporter), member 4 | 209610_s_at | -1.71 |
| PHLDA1 | pleckstrin homology-like domain, family A, member 1 | 225842_at | -1.76 |
| GRB14 | growth factor receptor-bound protein 14 | 206204_at | -1.82 |
| SORBS2 | sorbin and SH3 domain containing 2 | 225728_at | -1.84 |
| HIST1H1C | histone cluster 1, H1c | 209398_at | -1.85 |
| SLAMF7 | SLAM family member 7 | 234306_s_at | -1.91 |
| SEMA4A | sema domain, immunoglobulin domain (Ig), transmembrane domain (TM) and short cytoplasmic domain, (semaphorin) 4A | 234072_at | -1.95 |
| SLAMF7 | SLAM family member 7 | 219159_s_at | -1.99 |
| SLAMF7 | SLAM family member 7 | 222838_at | -1.99 |
| SEMA4A | sema domain, immunoglobulin domain (Ig), transmembrane domain (TM) and short cytoplasmic domain, (semaphorin) 4A | 219259_at | -2.41 |
| EDNRB | endothelin receptor type B | 204271_s_at | -2.78 |
| EDNRB | endothelin receptor type B | 206701_x_at | -3.10 |
| SERPINB9 | serpin peptidase inhibitor, clade B (ovalbumin), member 9 | 209723_at | -3.41 |
| EDNRB | endothelin receptor type B | 204273_at | -3.81 |

**Table S2A. Overlap of differentially expressed genes in LP-1/Cfz cells and genes downregulated in human lymphoblastoid cells treated with sulforaphane**

| **Gene Symbol** | **Gene Name** | **Probe Set ID** | **Fold Change** |
| --- | --- | --- | --- |
| HLA-DRB4 | major histocompatibility complex, class II, DR beta 4 | 209728_at | -15.22 |
| PTGER4 | prostaglandin E receptor 4 (subtype EP4) | 204897_at | -14.20 |
| HLA-DRB1/DRB3/DRB4 | major histocompatibility complex, class II, DR beta 1/DR beta 3/DR beta 4/DRB1-7 beta chain-like | 215193_x_at | -11.70 |
| HLA-DRB1/DRB4/DRB5 | major histocompatibility complex, class II, DR beta 1/DR beta 4/DR beta 5/DRB1-7 beta chain-like | 209312_x_at | -9.03 |
| CD74 | CD74 molecule, major histocompatibility complex, class II invariant chain | 1567628_at | -8.76 |
| HLA-DPB1 | major histocompatibility complex, class II, DP beta 1 | 201137_s_at | -8.41 |
| HLA-DRB1/HLA-DRB4 | major histocompatibility complex, class II, DR beta 1/DR beta 4/DRB1-7 beta chain-like | 204670_x_at | -8.04 |
| CD74 | CD74 molecule, major histocompatibility complex, class II invariant chain | 209619_at | -7.16 |
| HLA-DPA1 | major histocompatibility complex, class II, DP alpha 1 | 213537_at | -6.56 |
| HLA-DPA1 | major histocompatibility complex, class II, DP alpha 1 | 211991_s_at | -6.41 |
| FAM129A | family with sequence similarity 129, member A | 217967_s_at | -6.34 |
| FAM129A | family with sequence similarity 129, member A | 217966_s_at | -5.57 |
| HLA-DRB1/DRB3/  DRB4/DRB5 | major histocompatibility complex, class II, DR beta 1/DR beta 3/DR beta 4/DR beta 5/DRB1-7/DRB1-10 | 221491_x_at | -5.06 |
| BTN3A3 | butyrophilin, subfamily 3, member A3 | 38241_at | -4.20 |
| HLA-DPA1 | major histocompatibility complex, class II, DP alpha 1 | 211990_at | -4.06 |
| NUSAP1 | nucleolar and spindle associated protein 1 | 219978_s_at | -3.82 |
| ST8SIA4 | ST8 alpha-N-acetyl-neuraminide alpha-2,8-sialyltransferase 4 | 230836_at | -3.72 |
| KIAA0101 | KIAA0101 | 202503_s_at | -3.60 |
| PSAT1 | phosphoserine aminotransferase 1 | 223062_s_at | -3.31 |
| NUSAP1 | nucleolar and spindle associated protein 1 | 218039_at | -3.31 |
| BTN3A3 | butyrophilin, subfamily 3, member A3 | 204821_at | -3.29 |
| PSAT1 | phosphoserine aminotransferase 1 | 220892_s_at | -3.28 |
| CARD16/CASP1 | caspase recruitment domain family, member 16/caspase 1, apoptosis-related cysteine peptidase | 1552703_s_at | -3.27 |
| PAFAH1B3 | platelet-activating factor acetylhydrolase 1b, catalytic subunit 3 (29kDa) | 203228_at | -3.05 |
| KIAA0101 | KIAA0101 | 211713_x_at | -3.04 |
| CASP1 | caspase 1, apoptosis-related cysteine peptidase | 211368_s_at | -2.93 |
| BCAT1 | branched chain amino-acid transaminase 1, cytosolic | 226517_at | -2.84 |
| CASP1 | caspase 1, apoptosis-related cysteine peptidase | 206011_at | -2.67 |
| CASP1 | caspase 1, apoptosis-related cysteine peptidase | 209970_x_at | -2.63 |
| CASP1 | caspase 1, apoptosis-related cysteine peptidase | 211367_s_at | -2.61 |
| BCAT1 | branched chain amino-acid transaminase 1, cytosolic | 225285_at | -2.48 |
| ST8SIA4 | ST8 alpha-N-acetyl-neuraminide alpha-2,8-sialyltransferase 4 | 230261_at | -2.47 |
| CASP1 | caspase 1, apoptosis-related cysteine peptidase | 211366_x_at | -2.41 |
| ST8SIA4 | ST8 alpha-N-acetyl-neuraminide alpha-2,8-sialyltransferase 4 | 242943_at | -2.38 |
| BCAT1 | branched chain amino-acid transaminase 1, cytosolic | 214452_at | -2.14 |
| MYB | v-myb myeloblastosis viral oncogene homolog (avian) | 204798_at | -2.10 |
| MLKL | mixed lineage kinase domain-like | 238025_at | -2.10 |
| EPB41L2 | erythrocyte membrane protein band 4.1-like 2 | 201718_s_at | -2.04 |
| ADD3 | adducin 3 (gamma) | 205882_x_at | -2.03 |
| CENPF | centromere protein F, 350/400kDa | 207828_s_at | -2.00 |
| ADD3 | adducin 3 (gamma) | 201752_s_at | -2.00 |
| EPB41L2 | erythrocyte membrane protein band 4.1-like 2 | 201719_s_at | -1.99 |
| ID3 | inhibitor of DNA binding 3, dominant negative helix-loop-helix protein | 207826_s_at | -1.97 |
| SGK1 | serum/glucocorticoid regulated kinase 1 | 201739_at | -1.97 |
| IDH2 | isocitrate dehydrogenase 2 (NADP+), mitochondrial | 210046_s_at | -1.91 |
| PPP2R5C | protein phosphatase 2, regulatory subunit B', gamma | 214083_at | -1.90 |
| PTGER4 | prostaglandin E receptor 4 (subtype EP4) | 204896_s_at | -1.90 |
| ADD3 | adducin 3 (gamma) | 201753_s_at | -1.86 |
| SELL | selectin L | 204563_at | -1.85 |
| H2AFJ | H2A histone family, member J | 220936_s_at | -1.85 |
| CENPF | centromere protein F, 350/400kDa | 209172_s_at | -1.83 |
| NEIL3 | nei endonuclease VIII-like 3 (E. coli) | 219502_at | -1.83 |
| IVD | isovaleryl-CoA dehydrogenase | 225311_at | -1.81 |
| ST8SIA4 | ST8 alpha-N-acetyl-neuraminide alpha-2,8-sialyltransferase 4 | 206925_at | -1.80 |
| CALM1/CALM2/CALM3 | calmodulin 1 (phosphorylase kinase, delta)/calmodulin 2/calmodulin 3 | 200622_x_at | -1.79 |
| FBXO4 | F-box protein 4 | 223493_at | -1.77 |
| MKI67 | antigen identified by monoclonal antibody Ki-67 | 212022_s_at | -1.76 |
| HLA-DRB4 | major histocompatibility complex, class II, DR beta 4 | 215666_at | -1.76 |
| ALDH2 | aldehyde dehydrogenase 2 family (mitochondrial) | 201425_at | -1.75 |
| IVD | isovaleryl-CoA dehydrogenase | 216958_s_at | -1.75 |
| ADD3 | adducin 3 (gamma) | 201034_at | -1.74 |
| IDH2 | isocitrate dehydrogenase 2 (NADP+), mitochondrial | 210045_at | -1.72 |
| IVD | isovaleryl-CoA dehydrogenase | 203682_s_at | -1.70 |
| TUBB2A | tubulin, beta 2A class IIa | 204141_at | 1.74 |
| WHSC1 | Wolf-Hirschhorn syndrome candidate 1 | 222777_s_at | 1.74 |
| WHSC1 | Wolf-Hirschhorn syndrome candidate 1 | 223472_at | 1.94 |
| PTP4A3 | protein tyrosine phosphatase type IVA, member 3 | 206574_s_at | 2.10 |
| PTP4A3 | protein tyrosine phosphatase type IVA, member 3 | 209695_at | 2.14 |
| WHSC1 | Wolf-Hirschhorn syndrome candidate 1 | 222778_s_at | 2.30 |
| RGS20 | regulator of G-protein signaling 20 | 210138_at | 2.39 |
| S100A4 | S100 calcium binding protein A4 | 203186_s_at | 2.99 |
| PFKFB4 | 6-phosphofructo-2-kinase/fructose-2,6-biphosphatase 4 | 228499_at | 3.14 |

**Table S2B. Overlap of differentially expressed genes in KMS-11/Cfz cells and genes downregulated in human lymphoblastoid cells treated with sulforaphane**

| **Gene Symbol** | **Gene Name** | **Probe Set ID** | **Fold Change** |
| --- | --- | --- | --- |
| HLTF | helicase-like transcription factor | 202983_at | -4.09 |
| HMGCS1 | 3-hydroxy-3-methylglutaryl-CoA synthase 1 (soluble) | 205822_s_at | -2.45 |
| HIST1H2AC | histone cluster 1, H2ac | 215071_s_at | -2.20 |
| FADS1/MIR1908 | fatty acid desaturase 1/microRNA 1908 | 208962_s_at | -2.07 |
| HIST1H2BC/HIST1H2BE/  HIST1H2BF/HIST1H2BG/ HIST1H2BI | histone cluster 1, H2bc/histone cluster 1, H2be/histone cluster 1, H2bf/histone cluster 1, H2bg/histone cluster 1, H2bi | 214455_at | -2.05 |
| PFKFB4 | 6-phosphofructo-2-kinase/fructose-2,6-biphosphatase 4 | 228499_at | -2.02 |
| HIST2H2AA3/HIST2H2AA4 | histone cluster 2, H2aa3/histone cluster 2, H2aa4 | 218280_x_at | -2.00 |
| HLA-DPB1 | major histocompatibility complex, class II, DP beta 1 | 201137_s_at | -1.97 |
| HIST1H2AG/HIST1H2AH/  HIST1H2AI/HIST1H2AK/  HIST1H2AL/HIST1H2AM | histone cluster 1, H2ag/histone cluster 1, H2ah/histone cluster 1, H2ai/histone cluster 1, H2ak/histone cluster 1, H2al/histone cluster 1, H2am | 214554_at | -1.95 |
| HIST2H2BE | histone cluster 2, H2be | 202708_s_at | -1.91 |
| HIST1H2BH | histone cluster 1, H2bh | 208546_x_at | -1.91 |
| FADS1/MIR1908 | fatty acid desaturase 1/microRNA 1908 | 208964_s_at | -1.89 |
| HIST1H2BC | histone cluster 1, H2bc | 236193_at | -1.88 |
| HIST1H1C | histone cluster 1, H1c | 209398_at | -1.85 |
| CYSLTR1 | cysteinyl leukotriene receptor 1 | 230866_at | -1.82 |
| HIST2H2AA3/HIST2H2AA4 | histone cluster 2, H2aa3/histone cluster 2, H2aa4 | 214290_s_at | -1.82 |
| HIST1H2BD | histone cluster 1, H2bd | 209911_x_at | -1.76 |
| HMGCS1 | 3-hydroxy-3-methylglutaryl-CoA synthase 1 (soluble) | 221750_at | -1.74 |
| CYSLTR1 | cysteinyl leukotriene receptor 1 | 231747_at | -1.72 |
| HIST1H2BC/HIST1H2BE/  HIST1H2BF/HIST1H2BG/  HIST1H2BI | histone cluster 1, H2bc/histone cluster 1, H2be/histone cluster 1, H2bf/histone cluster 1, H2bg/histone cluster 1, H2bi | 208527_x_at | -1.72 |
| APOL3 | apolipoprotein L, 3 | 221087_s_at | -1.71 |
| HIST1H2BC/HIST1H2BE/  HIST1H2BF/HIST1H2BG/  HIST1H2BI | histone cluster 1, H2bc/histone cluster 1, H2be/histone cluster 1, H2bf/histone cluster 1, H2bg/histone cluster 1, H2bi | 208490_x_at | -1.68 |
| DDIT4 | DNA-damage-inducible transcript 4 | 202887_s_at | -1.66 |
| HIST1H2BD | histone cluster 1, H2bd | 222067_x_at | -1.66 |
| HIST1H2AB/HIST1H2AE | histone cluster 1, H2ab/histone cluster 1, H2ae | 214469_at | -1.63 |
| PHGDH | phosphoglycerate dehydrogenase | 201397_at | -1.62 |
| LIMA1 | LIM domain and actin binding 1 | 217892_s_at | -1.61 |
| RGS20 | regulator of G-protein signaling 20 | 210138_at | -1.60 |
| LCK | lymphocyte-specific protein tyrosine kinase | 204891_s_at | -1.60 |
| WIPI1 | WD repeat domain, phosphoinositide interacting 1 | 203827_at | -1.59 |
| H2AFJ | H2A histone family, member J | 225245_x_at | -1.56 |
| WIPI1 | WD repeat domain, phosphoinositide interacting 1 | 213836_s_at | -1.55 |
| GATM | glycine amidinotransferase (L-arginine:glycine amidinotransferase) | 203178_at | -1.55 |
| H2AFJ | H2A histone family, member J | 224301_x_at | -1.54 |
| RAD54B | RAD54 homolog B (S. cerevisiae) | 219494_at | -1.54 |
| HIST1H2BC/HIST1H2BE/  HIST1H2BF/HIST1H2BG/  HIST1H2BI | histone cluster 1, H2bc/histone cluster 1, H2be/histone cluster 1, H2bf/histone cluster 1, H2bg/histone cluster 1, H2bi | 208523_x_at | -1.51 |
| GATM | glycine amidinotransferase (L-arginine:glycine amidinotransferase) | 216733_s_at | -1.51 |
| ALDOC | aldolase C, fructose-bisphosphate | 202022_at | -1.50 |
| HIST1H2BK | histone cluster 1, H2bk | 209806_at | -1.50 |
| DHCR24 | 24-dehydrocholesterol reductase | 200862_at | -1.49 |
| HIST1H3H | histone cluster 1, H3h | 206110_at | -1.47 |
| HIST1H2AD/HIST1H3A/  HIST1H3B/HIST1H3C/  HIST1H3D/HIST1H3E/  HIST1H3F/HIST1H3G/  HIST1H3H/HIST1H3I/  HIST1H3J | histone cluster 1, H2ad/histone cluster 1, H3a/histone cluster 1, H3b/histone cluster 1, H3c/histone cluster 1, H3d/histone cluster 1, H3e/histone cluster 1, H3f/histone cluster 1, H3g/histone cluster 1, H3h/histone cluster 1, H3i/histone cluster 1, H3j | 214522_x_at | -1.46 |
| SCD | stearoyl-CoA desaturase (delta-9-desaturase) | 211708_s_at | -1.46 |
| SCD | stearoyl-CoA desaturase (delta-9-desaturase) | 211162_x_at | -1.46 |
| LCK | lymphocyte-specific protein tyrosine kinase | 204890_s_at | -1.44 |
| SP140 | SP140 nuclear body protein | 207777_s_at | -1.44 |
| HIST1H2BJ | histone cluster 1, H2bj | 214502_at | -1.44 |
| SKP2 | S-phase kinase-associated protein 2, E3 ubiquitin protein ligase | 210567_s_at | -1.43 |
| BFSP2 | beaded filament structural protein 2, phakinin | 207399_at | -1.42 |
| HIST1H2AM | histone cluster 1, H2am | 214481_at | -1.40 |
| INSIG1 | insulin induced gene 1 | 201625_s_at | -1.39 |
| HIST1H2AD/HIST1H3A/  HIST1H3B/HIST1H3C/  HIST1H3D/HIST1H3E/  HIST1H3F/HIST1H3G/  HIST1H3H/HIST1H3I/  HIST1H3J | histone cluster 1, H2ad/histone cluster 1, H3a/histone cluster 1, H3b/histone cluster 1, H3c/histone cluster 1, H3d/histone cluster 1, H3e/histone cluster 1, H3f/histone cluster 1, H3g/histone cluster 1, H3h/histone cluster 1, H3i/histone cluster 1, H3j | 214472_at | -1.38 |
| HIST1H4A/HIST1H4B/  HIST1H4C/HIST1H4D/  HIST1H4E/HIST1H4F/  HIST1H4H/HIST1H4I/  HIST1H4J/HIST1H4K/  HIST1H4L/HIST2H4A/  HIST2H4B/HIST4H4 | histone cluster 1, H4a/histone cluster 1, H4b/histone cluster 1, H4c/histone cluster 1, H4d/histone cluster 1, H4e/histone cluster 1, H4f/histone cluster 1, H4h/histone cluster 1, H4i/histone cluster 1, H4j/histone cluster 1, H4k/histone cluster 1, H4l/histone cluster 2, H4a/histone cluster 2, H4b/histone cluster 4, H4 | 208180_s_at | -1.37 |
| IDH1 | isocitrate dehydrogenase 1 (NADP+), soluble | 1555037_a_at | -1.37 |
| SLCO4C1 | solute carrier organic anion transporter family, member 4C1 | 222071_s_at | -1.36 |
| CGN | cingulin | 223232_s_at | -1.36 |
| SQLE | squalene epoxidase | 213577_at | -1.36 |
| SQLE | squalene epoxidase | 213562_s_at | -1.36 |
| ANXA2 | annexin A2 | 201590_x_at | 1.42 |
| PTGER4 | prostaglandin E receptor 4 (subtype EP4) | 204897_at | 1.43 |
| EID3 | EP300 interacting inhibitor of differentiation 3 | 231292_at | 1.45 |
| ANXA2 | annexin A2 | 210427_x_at | 1.46 |
| FAM129A | family with sequence similarity 129, member A | 217967_s_at | 1.46 |
| ANXA2 | annexin A2 | 213503_x_at | 1.49 |
| GBP1 | guanylate binding protein 1, interferon-inducible | 231577_s_at | 1.50 |
| GBP1 | guanylate binding protein 1, interferon-inducible | 202270_at | 1.53 |
| GBP1 | guanylate binding protein 1, interferon-inducible | 202269_x_at | 1.55 |
| ALOX5 | arachidonate 5-lipoxygenase | 204446_s_at | 1.66 |
